# Polypyrimidine tract-binding proteins of potato mediate tuberization through an interaction with *StBEL5* RNA

**DOI:** 10.1093/jxb/erv389

**Published:** 2015-08-17

**Authors:** Sung Ki Cho, Pooja Sharma, Nathaniel M. Butler, Il-Ho Kang, Shweta Shah, A. Gururaj Rao, David J. Hannapel

**Affiliations:** ^1^Plant Biology Major, Iowa State University, Ames, IA 50011-1100, USA; ^3^Department of Biochemistry, Biophysics and Molecular Biology, Iowa State University, Ames, IA 50011, USA

**Keywords:** Mobile RNA, phloem, RNA-binding protein, *Solanum tuberosum*, StPTB, tuberization

## Abstract

Polypyrimidine tract-binding (PTB) proteins are a family of RNA-binding proteins that function in a wide range of RNA metabolic processes by binding to motifs rich in uracils and cytosines. A PTB protein of pumpkin was identified as the core protein of an RNA–protein complex that trafficks RNA. The biological function of the PTB–RNA complex, however, has not been demonstrated. In potato, six PTB proteins have been identified, and two, designated StPTB1 and StPTB6, are similar to the phloem-mobile pumpkin type. RNA binding assays confirmed the interaction of StPTB1 and StPTB6 with discrete pyrimidine-rich sequences of the 3′-untranslated regions of the phloem-mobile mRNA, *StBEL5*. The promoter of *StPTB1* was active in companion cells of phloem in both stem and petioles. Expression of both types was evident in phloem cells of roots and in stolons during tuber formation. RNA accumulation of both PTB proteins was induced by short days in leaves in correlation with enhanced accumulation of *StBEL5* RNA. StPTB suppression lines exhibited reduced tuber yields and decreased *StBEL5* RNA accumulation, whereas StPTB overexpression lines displayed an increase in tuber production correlated with the enhanced production in stolons of steady-state levels of *StBEL5* transcripts and RNA of key tuber identity genes. In StPTB overexpression lines, both the stability and long-distance transport of *StBEL5* transcripts were enhanced, whereas in suppression lines stability and transport decreased. Using a transgenic approach, it is shown that the StPTB family of RNA-binding proteins regulate specific stages of development through an interaction with phloem-mobile transcripts of *StBEL5*.

## Highlight

PTB proteins of potato bind to the mobile RNA, *StBEL5*, and enhance stability and trafficking of the RNA to select organs. This protein–RNA interaction leads to enhanced tuber production.

## Introduction

The polypyrimidine tract-binding (PTB) family of proteins represent a multifaceted group of proteins that binds numerous mRNAs and have been implicated in a wide range of RNA metabolic processes including stability (Xu and Hecht, 2007), splicing regulation (Valcarcel and Gebauer, 1997; Xue *et al.*, 2009), intracellular localization (Kuwahata *et al.*, 2007), translation repression (Karakasiliotis *et al.*, 2010), and control of long-distance transport (Ham *et al.*, 2009). PTBs have also been implicated in alternative splicing (AS) of their own pre-mRNAs (Wollerton *et al.*, 2004). PTBs bind viral, mammalian, and plant RNAs with binding sites rich in uracils and cytosines. The structure of a PTB protein is uniquely adapted to these multiple functions. They generally contain four RNA-recognition motifs (RRMs) of ~90 amino acids connected by linker regions and designated RRM1, RRM2, RRM3, and RRM4. Each RRM is formed by 4–5 β-sheets and contains 6–8 conserved amino acids, designated RNP1 and RNP2, that interact with CU (cytosine uracil) motifs, ranging from three to five nucleotides in length (Oberstrass *et al.*, 2005; Auweter and Allain, 2008). The four RRMs of human PTB, hnPTB1, recognize 3, 4, 5, and 3 nucleotides, respectively (Auweter and Allain, 2008). Structural analysis has revealed that RRM1 and RRM2 are linear and function independently, whereas RRM3 and RRM4 act in a tandem, compact complex that functions as an open-faced clamp on closely spaced polypyrimidine tract motifs (Oberstrass *et al.*, 2005).

Despite their widespread function and versatility in eukaryotes, very little information is available on the role of PTB proteins in plants. There are three PTB genes in *Arabidopsis*, two of which are involved in pollen tube development (Wang and Okamoto, 2009). The three *PTB* genes from *Arabidopsis* auto- and cross-regulate their expression through alternative splicing coupled to nonsense-mediated decay (Stauffer *et al.*, 2010). Clearly, some plant PTBs play an important role in regulating AS (Wachter *et al.*, 2012). A transcriptome-wide analysis in transgenic lines that under- and overexpressed AtPTBs and a mini-exon splicing reporter system revealed that both AtPTB1 and AtPTB2 were involved in regulating AS that impacted numerous developmental processes (Rühl *et al.*, 2012; Simpson *et al.*, 2014). No significant AS regulatory function, however, was observed for the distantly related AtPTB3. AtPTB1 and AtPTB2 contain three RRMs, whereas AtPTB3 contains four. This latter PTB is closely related to the pumpkin PTB protein, RBP50. Ham *et al.* (2009) demonstrated that RBP50 was the core protein of a phloem-mobile RNA–protein complex consisting of 16 proteins and six RNAs. Included in this group were full-length transcripts for *PHLOEM PROTEIN16* (*PP16-1*), *GIBBERELLIC ACID-INSENSITIVE* (*GAI*), a *SCARECROW-LIKE* protein, *SHOOT MERISTEMLESS*, an *ETHYLENE RESPONSE FACTOR*, and a *MYB* transcription factor. Gel mobility shift assays confirmed that specific binding of CmRBP50 to two of its target RNAs, *GAI* and *PP16-1* of pumpkin, was facilitated by polypyrimidine tract motifs located within the transcript sequences (Ham *et al.*, 2009). PTB proteins are ubiquitous in the plant kingdom, and as RNA chaperones some types of PTB proteins appear to play a critical role beyond a splicing repressor function in protecting and mobilizing full-length mRNAs that are transported through the phloem. Despite important information on the biochemistry of these mobile RNA-binding proteins, however, very little is known about their biological function in whole-plant systems. As an example, virtually nothing is known about the function of AtPTB3.

Through functional and genomic analyses, the PTB family of potato (*Solanum tuberosum*), represented by six genes, two containing four RRMs and designated *StPTB1* and *StPTB6*, and four three-RRM types, have been characterized. Based on amino acid sequence and function, StPTB1 and StPTB6 appear to be orthologues of AtPTB3 and CmRBP50, and serve as chaperones to full-length mRNAs that are transported through the sieve element system. RNA binding assays confirmed the interaction of StPTB1 and StPTB6 with the 3′-untranslated regions (UTRs) of the mobile RNA, *StBEL5*. In novel analyses using transgenic whole-plant systems, it is demonstrated that PTB proteins of potato enhance stability and regulate transport of a mobile RNA and specifically control tuber development.

## Materials and methods

### Identification and cloning of cDNA sequences

To obtain the full-length *StPTB* and *StGAI* cDNA sequences, the DFCI Gene Index potato database (http://www.danafarber.org) was searched using the *CmRBP50* and *CmGAI* sequence as query, respectively. From partial cDNA sequences, the rapid amplification of cDNA ends (RACE) was performed using the First Choice RLM-RACE kit (Ambion) to identify the 5′- and 3′-untranslated sequences. Full-length cDNA sequences were then obtained by PCR amplification with 5′ and 3′ gene-specific primers from RACE experiments. The amplified PCR fragments were cloned into pCRII-TOPO vector (Invitrogen) and verified by sequencing. *StPTB1*, *StPTB6*, and *StPTB7* were identified in this manner. The primers used in this study (Supplementary Table S1 available at *JXB* online) were synthesized at the DNA Facility, Iowa State University.

### RNA analysis

Transgenic and wild-type plants of *S. tuberosum* cv. Désirée were used for most of the studies reported here. The photoperiod-responsive potato (*S. tuberosum* ssp*. andigena*, line 7540) was used for the quantitative reverse transcription–PCR (qRT–PCR) and protein analyses. RNA extraction and qRT–PCR were performed as previously described (Lin *et al.*, 2013). Gene-specific primers used for the quantitative qRT–PCR are listed in Supplementary Table S3 at *JXB* online.

### Constructs

For the Prom_StPTB1_:GUS (β-glucuronidase) and Prom_StPTB6_:GUS plasmids, the constructs include DNA fragments containing 2519bp and 3593bp of sequence upstream of the translational start codon, respectively (Butler and Hannapel, 2012). Promoter fragments were obtained by PCR amplification using the primers listed in Supplementary Table S4 at *JXB* online and cloned into pBI101.1-GUS (Clontech). For transformation of the 35S:StPTB–GFP (green fluorescent protein) constructs, the pBI121-GFP vector was generated from pBI121-GUS (Clontech) by replacing the GUS gene with the GFP reporter gene in *Bam*HI/*Sac*I sites in the pBI121-GUS vector. The 35S:StPTB1–GFP and 35S:StPTB6–GFP constructs include the coding sequences of StPTB1 and StPTB6 without the stop codon and fused in-frame to GFP. The inserts for the constructs were obtained by PCR amplification and directly cloned into the pBI121-GFP vector (Supplementary Table S4).

### RNA suppression lines for *StPTB1* and *StPTB6*

To suppress *StPTB1* and *StPTB6* transcript levels, both antisense and RNAi constructs were designed. The antisense construct was made from the 611bp *Sac*I–*Spe*I fragment of the *StPTB6* cDNA (87% identical to the *StPTB1* cDNA sequence), cloned in the antisense direction into the binary vector, pCB201 (Xiang *et al.*, 1999), and driven by the *Cauliflower mosaic virus* (CaMV) 35S promoter. For generating the RNAi construct (Supplementary Table S1 at *JXB* online), the same conserved 611bp fragment of the *StPTB6* cDNA was cloned into the pENTR/D-TOPO plasmid (Invitrogen) and inserted in opposite orientations by recombination with the LR Clonase II enzyme (Gateway Technology, Invitrogen) into the pBIN19RNAi destination vector (Navarro *et al.*, 2011). Transformation was undertaken on leaf sections from *S. tuberosum* cv. Désirée as described by Banerjee *et al.* (2006*a*). Functional transformants were screened for a reduction in the level of transcripts of both *StPTB1* and *StPTB6* in RNA samples from leaves and stolons.

### Histological analysis of promoter activity

Select transgenic promoter lines were transferred to soil and grown for 2 weeks before tissues were harvested and stained for 16h using a 1.0mg ml^–1^ X-gluc (5-bromo-4-chloro-3-indolyl-β-d-GlcUA) solution and cleared using 70% ethanol. Fixation of stained materials (Fig. 2) was performed with FAA (10% formaldehyde, 50% ethanol, 5% acetic acid) using vacuum infiltration and dehydrated through an ethanol series for embedding in paraffin. Prepared paraffin sections of ~10 μm were fixed to glass slides for microscopy and photodocumented using an Axioplan compound light microscope with a colour camera mount.

### Preparation of polyclonal antibody

*StPTB1* and *StPTB6* cDNA inserts were cloned into the pET-28a (+) plasmid and transformed into *Escherichia coli* BL21-Codon (DE3) cells (Stratagene, La Jolla, CA, USA). After induction with 0.4mM isopropyl-β-d-thiogalactopyranoside (IPTG), the recombinant proteins were purified using Ni-NTA agarose (Qiagen, Valencia, CA, USA) and quantified. These purified proteins were used for EMSA and for preparation of polyclonal antibody. Polyclonal antibodies against StPTB6 protein were raised at the Hybridoma Facility at Iowa State University. The purified proteins (500–800 μg) were repeatedly injected into two rabbits, and standard protocols were used for bleeding and screening to obtain reliable antibodies. The sera containing specific polyclonal antibodies against StPTB6 were used for the studies without purification. Pre-immune controls are shown in Supplementary Fig. S6 at *JXB* online.

### Electrophoretic mobility shift assays

For RNA-EMSAs, target sequences were amplified from potato leaf cDNA or genomic DNA using corresponding primers (Supplementary Table S4 at *JXB* online). These probes were derived from either coding sequence or the 3′-UTR, and were selected based on the yeast three-hybrid results (Supplementary Fig. S4). RNA bait was generated using *in vitro* transcription with the MAXIscript^®^ T3 kit (Ambion) and biotin-labeled UTP (Bio-11-UTP, Ambion). Biotin-labelled RNA probes were purified using RNA Clean & Concentrator™-5 (Zymo Research) and quantified by NanoDrop 1000 (Thermo Scientific). Biotin-labelled RNAs (3fmol) were used for the binding assay. Binding reactions with labelled RNA and purified recombinant proteins (0–500nM) were incubated in 20 µl for 1.0h at room temperature in the presence of a binding buffer consisting of 40mM Tris (pH 8.0), 30mM KCl, 1mM MgCl_2_, 0.1% NP-40, and 1mM DTT. The binding reactions were resolved on a 6% (v/v) non-denaturing polyacrylamide gel, and then transferred onto a BrightStar^®^-Plus positively charged nylon membrane (Ambion). For detection of biotinylated RNA, the Chemiluminescent nucleic acid detection module kit (Thermo Scientific) and CL-XPosure™ film (Thermo Scientific) were used.

### RNA immunoprecipitation

New tubers (~4.0–8.0mm in diameter) or petioles of *S. tuberosum* ssp. *andigena* plants grown under short-day (SD) conditions were fixed using 1.0% formaldehyde and 125mM glycine under a vacuum. The fixed samples were frozen and ground in liquid nitrogen, and total cell extracts were prepared with 0.7g of tissue sample in 2.0ml of RIP-LB buffer (Köster and Staiger, 2014), and cleared using Dynabeads^®^ Protein A (Invitrogen). For immunoprecipitation (IP), 20 µl of Dynabeads^®^ Protein A were coated with 2.0 µg of pre-immune serum, αStPTB6 antibody purified using the Melon Gel IgG purification kit (Thermo Scientific), or αGFP monoclonal antibody (Santa Cruz), and washed as previously described (Terzi and Simpson, 2009). For IP of *StBEL5* RNA and StPTBs proteins, 600 µl of pre-cleared total cell extracts using a 0.2 µm syringe filter (VWR) were incubated with antibody-coated magnetic beads for 2h at 4 °C under rotation. After intensive washings (Terzi and Simpson, 2009), to release RNAs that were cross-linked with StPTB proteins by formaldehyde fixation, NaCl was added to 200mM final concentration, and IP reactions were incubated for 20min at 65 °C.

Diluted input (1.0%) of purified RNA from the IP was used for gel-based RT–PCR. For qRT–PCR, the qScript™ One-Step SYBR^®^ Green qRT-PCR kit (Quanta Biosciences) with the Eco Real-Time PCR system (Illumina) was used. Each real-time PCR was performed in duplicate. For quantification and comparison of RNA levels between samples, the 2^−ΔΔCt^ method was used (Livak and Schmittgen, 2001). To account for RNA sample preparation differences, each RIP RNA fraction’s Ct value was normalized to 1.0% of the input RNA fraction Ct value for each corresponding qPCR assay according to the RIP protocol (Sigma, Imprint^®^ RNA RIP Kit). Primer sequences used are listed in Supplementary Table S5 at *JXB* online.

### *Potato virus X* (PVX) vector system for RNA movement assay

Full-length cDNAs, including the 5′- and 3′-UTRs, of *StBEL5* and *StBEL14* were amplified using NEBNext High-Fidelity 2X PCR Master Mix (NEB), and cloned into *Mlu*I and *Eco*RV sites of the PVX/ΔCP vector (Li *et al.*, 2009) to create PVX/B5H and PVX/B14H, respectively. The control PVX/GFP vectors were described previously by Li *et al.* (2009). Reverse primers for the *StBEL* constructs were designed to include a histidine-tag sequence for inoculum-specific detection (Supplementary Table S5 at *JXB* online). Transcripts for mobility assays were prepared by *in vitro* transcription using a T7 High Yield RNA Synthesis kit (NEB), as described in the instruction manual, and purified using LiCl precipitation. A 10 µg aliquot of *in vitro* transcripts was mechanically inoculated onto each leaf (2–3 leaves per plant) of wild-type *S. tuberosum* cv Désirée or the StPTB Désirée transgenic lines. Inoculated leaves, and non-inoculated roots and stolons were harvested at 8 d post-inoculation, and total RNA was extracted using an RNeasy Plant Mini kit (Qiagen) and followed by RNase-free DNase (Qiagen) treatment. Total RNA (150ng) was used for the gel-based RT–PCR. For qRT–PCR, the qScript One-Step SYBR Green qRT-PCR kit (Quanta Biosciences) with the Eco Real-Time PCR system (Illumina) was used. After normalization using *StAct8* as an endogenous control, the relative gene quantification approach (Livak and Schmittgen, 2001) was applied to calculate the mobility level of the mechanically inoculated *StBEL5*, and analysed using a Student’s *t*-test. Levels of RNA in roots and stolons were calculated in relation to the amount of leaf inoculum.

## Results

### The PTB proteins of potato

Using RT–PCR and 5′ and 3′ RACE, three full-length RNAs, designated *StPTB1* (1487 nucleotides), *StPTB6* (1624 nucleotides), and *StPTB7* (1678 nucleotides), were identified that encode for StPTB proteins. The lengths of these three proteins are 441, 444, and 467 amino acids, respectively. StPTB1 and StPTB6 closely match the amino acid sequence of the PTB protein of pumpkin, CmRBP50 (Supplementary Fig. S1 at *JXB* online). Each of the proteins contains conserved RRMs, the conventional set of four for StPTB1 and StPTB6 but only three for StPTB7 (a three-RRM type). StPTB7 contains 11 extra residues at the N-terminus and lacks a conserved RRM4 (Supplementary Fig. S1). The conserved motifs of the RRM (Auweter and Allain, 2008; Cléry *et al.*, 2008), consisting of eight amino acids that interact with target RNAs, RNP1 and RNP2, are present in each of the identified RRMs (Supplementary Fig. S1, underlined sequence). One other StPTB protein has been identified from the DFCI Potato Gene Index (TC201749) and two others from the potato genome (BI920231 and TC218925; Xu *et al.*, 2011). Each of these latter three is closely related to StPTB7 (Supplementary Fig. S2). Based on phylogenetic analysis (Supplementary Fig. S3), StPTB7 and these other three-RRM types belong to the same clade with AtPTB1 and AtPTB2 (Wang and Okamoto, 2009). Phylogenetic analysis classifies the PTBs of plants into two categories, one designated the StPTB1/PTB6 (CmRBP50-like) clade and a second designated the StPTB7 clade (Supplementary Fig. S3). Because of our interest in phloem-mobile mRNAs (Banerjee *et al.*, 2006*b*), the focus of this current study is the CmRBP50 types in potato, StPTB1 and StPTB6 (Supplementary Fig. S2).

### RNA binding properties of the StPTB proteins

**Fig. 1.**
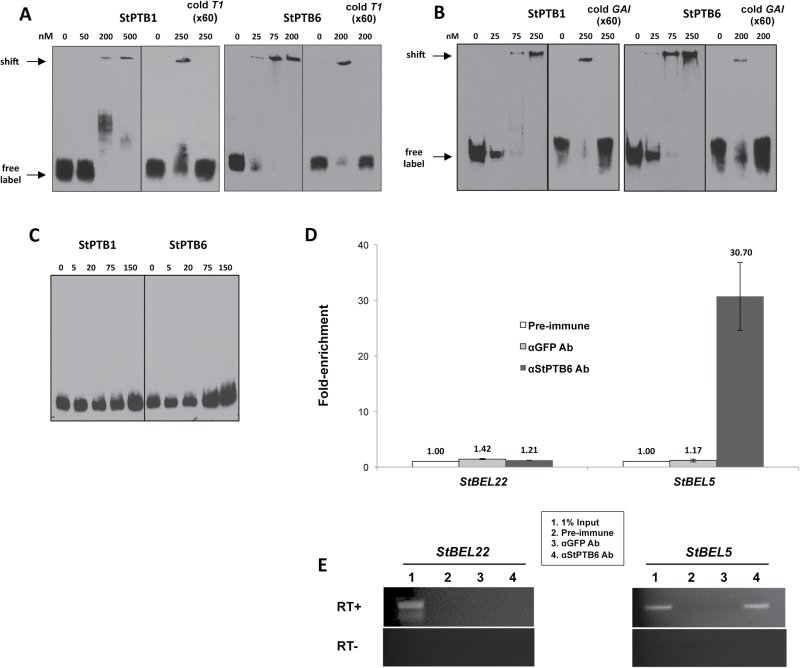
RNA binding assays using RNA gel shifts (A–C) and RNA immunoprecipitation (D, E). For gel-shift assays, select RNA with the PTB1 and PTB6 proteins of potato were utilized (A–C). Labelled RNA included the 137 nucleotide *T1* subset sequence of the *StBEL5* 3′-UTR (A), a 77 nucleotide 3′-UTR sequence from *StGAI* (B), and a 101 nucleotide coding sequence, *StBEL5-N*, as a negative control (C). The complete sequence of these three RNA baits is shown in Supplementary Fig. S8 at *JXB* online. Unlabelled *T1* or *GAI* probe to 60-fold excess was used with 200nM (StPTB6) or 250nM (StPTB1) of protein in competition assays for each interaction shown in the last three lanes of each StPTB panel (A, B). For these three lanes: 0 lane is label without protein, the 200 or 250 lane is label plus protein, and cold *T1* or *GAI* (×60) lanes are the competition assays containing both unlabelled and labelled probe. RNA bait was labelled with biotin using *in vitro* transcription and then incubated with purified StPTB proteins in concentrations ranging from 5nM to 500nM protein. Approximately 3.0fmol of labelled bait RNA was used in each binding reaction. RNA immunoprecipitation of *StBEL5* RNA was performed from new tubers using αStPTB6 antibody. New tubers of *S. tuberosum* ssp. *andigena* plants grown under short-day conditions for 14 d were used as the input sample. For quantification and comparison of RNA levels between samples (D), the 2^–∆∆Ct^ method was used (Livak and Schmittgen, 2001). Each real-time PCR was performed in duplicate. To account for RNA sample preparation differences, each RIP RNA fraction’s Ct value was normalized to 1.0% of the input RNA fraction Ct value for the same qPCR assay. RNA for *StBEL22* was quantified in parallel with *StBEL5* and used as a negative control. αGFP monoclonal antibody was used as a negative immunoprecipitation control. Quantitative RT–PCR values (D) were normalized to the levels in the pre-immune sample. RNAs from the immunoprecipitations and 1.0% diluted input were used for gel-based RT–PCR with (+) and without (–) reverse transcriptase (E).

To test their putative function as chaperones to phloem-mobile RNAs, the ability of StPTB1 and StPTB6 to bind to RNA sequence was assessed. Preliminary analysis utilizing the yeast three-hybrid system suggested that StPTB1 and StPTB6 proteins interacted with specific 3′-UTR RNA sequence of both *StBEL5* (*T1*) and *StGAI* (Supplementary Fig. S4 at *JXB* online). To verify protein–RNA interactions, RNA gel-shift assays with StPTB1 and StPTB6 were performed (Fig. 1A–C). Both proteins generated a shifted band in an interaction with both the labelled *StBEL5 T1* and *StGAI* fragments (bait sequences for both types are shown in Supplementary Fig. S5). For the *T1* bait, an interaction was observed for StPTB6 with a concentration as low as 25nM (Fig. 1A), whereas StPTB1 affected a shift at higher protein concentrations (Fig. 1A). For the *StGAI* bait, an interaction was observed for StPTB6 with a concentration as low as 25nM (Fig. 1B), whereas StPTB1 affected a shift at 75nM (Fig. 1B). No band shift was evident for either protein with a negative control interaction using *StBEL5-N* as bait (Fig. 1C) and even with protein concentrations up to 300nM (not shown). It should be noted that the negative control used in this gel shift contained no major CU motifs, whereas the *StGAI* and *T1* baits each contains five CU motifs (Supplementary Fig. S5). Results with both the yeast three-hybrid and the RNA gel-shift assays suggest that the interaction of StPTB6 with both *T1* and the *StGAI* UTR fragments was stronger than the interaction with StPTB1 under the current experimental conditions. RNA IP confirms that StPTB proteins bind to *StBEL5* RNA in planta (Fig. 1D, E). Crude protein–RNA extracts from new tubers were fixed with formaldehyde and incubated with αStPTB6 antibody (Supplementary Figs S6, S7). Immunoprecipitation of the new tuber extract with the StPTB6 antibody yielded a 30-fold enrichment of *StBEL5* RNA relative to the control sample (Fig. 1D). No transcript enrichment was observed for the negative control, full-length *StBEL22*. *StBEL22* was used here because its 74 nucleotide 3′-UTR contained no CU motifs longer than 3 nucleotides (Supplementary Fig. S5). These results were reproducible in both replicate tuber- and petiole-derived fractions.

### Expression patterns of *StPTB* genes

**Fig. 2.**
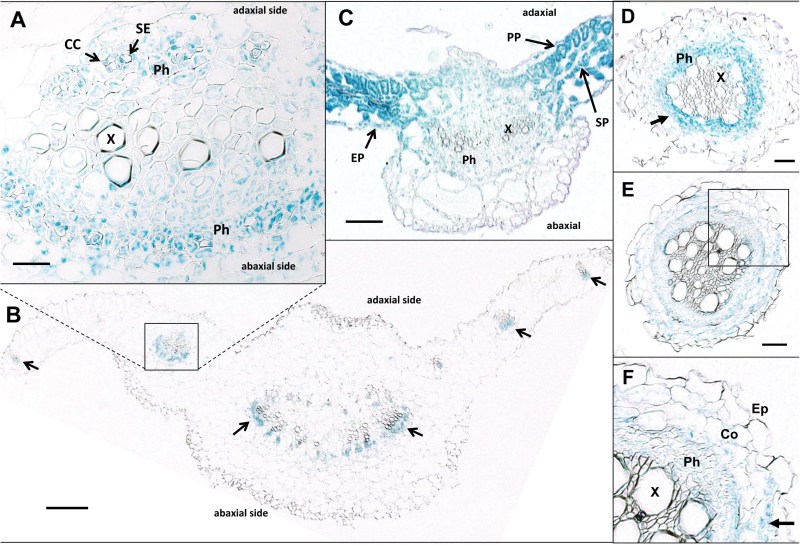
Localization of GUS activity within petioles and leaves of *StPTB1*_*prom*_ (A, B) and *StPTB6*_*prom*_ (C) transgenic lines. Petioles from 4-week-old soil-grown plants of *StPTB1*_*prom*_ (A, B) and leaves of 2-week-old *in vitro* plants of *StPTB6*_*prom*_ (C) were embedded in paraffin for histochemical detection of GUS activity within petiole and leaf tissues. (A) A transverse section of *StPTB1*_*prom*_ with a higher magnification image of a petiole vascular bundle comparable with the boxed region of (B) showing xylem (X) and phloem (Ph) tissues. The arrows in (B) indicate GUS activity of the *StPTB1*_*prom*_ in abaxial-side phloem cells. (C) A transverse section of *StPTB6*_*prom*_ showing the midvein and adjacent leaf blades with strong staining in the epidermis (EP), palisade (PP), and spongy (SP) parenchyma tissues. The scale bars represent 20 μm in (A), 200 μm in (B), and 100 μm in (C). CC, companion cell; SE, sieve element; Ph, phloem; X, xylem; EP, epidermis; PP, phloem parenchyma; SP, spongy parenchyma. GUS activity within a transverse section of primary roots of *StPTB1*_*prom*_ (D) and *StPTB6*_*prom*_ (E, F) transgenic lines. *StPTB1*_*prom*_ activity was observed in cells of the vascular core, mostly in phloem cells (D, arrow), whereas *StPTB6*_*prom*_ activity was dispersed throughout the phloem and cortex tissues (E, F). Staining was observed in the phloem (Ph) and cortex (Co), but not in the epidermis (Ep) or xylem (X). (F) A higher magnification of (E) (box). The scale bars represent 0.1mm in (D and E). The arrow in (F) designates GUS activity in cortical cells.

To determine if *StPTB* promoter activity coincides with *StBEL5* expression, *StPTB1* and *StPTB6* activity was assayed in transgenic potato lines (cv. Désirée) by fusing ~3.0kb of upstream sequence of both types to a GUS marker. *StBEL5* promoter activity was observed in leaf veins, petioles, and in phloem cells of the petiole (Banerjee *et al.*, 2006*b*). *StPTB1* and *StPTB6* exhibited promoter activity in stems, petioles, and leaf veins (Butler and Hannapel, 2012). For a more detailed look, activity within leaf tissues was examined in transverse sections of petioles with attached leaf blades (Fig. 2). *StPTB1*_*prom*_ activity was consistently observed throughout both adaxial and abaxial phloem of petioles (Fig. 2A, B, arrows). Specific *StPTB1*_*prom*_ activity can be observed in companion cells (CCs) but not sieve elements (SEs) of petiole phloem (Fig. 2A, arrows). In leaves, *StPTB6*_*prom*_ activity was not localized to vascular cells of the midvein (Fig. 2C, X and Ph), but was widespread in phloem (Fig. 2C, Ph), the epidermis (EP), and parenchyma (PP, SP) of adjacent leaf blades (Fig. 2C, arrows). In transverse sections of roots, *StPTB1*_*prom*_ activity was observed predominantly in the phloem (Fig. 2D, arrow), whereas *StPTB6*_*prom*_ activity was observed in both phloem and cortical cells (Fig. 2E, F, arrow). In general, in stems, leaves, and roots, the *StPTB1* promoter was most active in phloem cells. The *StPTB6* promoter exhibited activity in the phloem of roots but overall more activity than *StPTB1* in ground tissues of these organs.

Transverse sections of stained internodes from stems of *StPTB1*_*prom*_ and *StPTB6*_*prom*_ lines were also visualized (Supplementary Fig. S8A at *JXB* online). Discrete staining was observed for the *StPTB1*_*prom*_ line throughout the external phloem (EP, Supplementary Fig. S8B) layers, with some staining within the internal phloem (IP, Supplementary Fig. S8B). The activity of the *StPTB1*_*prom*_ was specific to CCs but absent from SEs in the vascular bundles of these stem sections (Supplementary Fig. S8C, D). In contrast, *StPTB6*_*prom*_ activity was more widespread and irregular in the stem. *StPTB6*_*prom*_ activity was not as abundant in vascular bundles of stems as the *StPTB1*_*prom*_ construct (Supplementary Fig. S8E, F), but was observed in some cells of the internal phloem (IP, Supplementary Fig. S8F), in interfascicular regions, and in clusters of cells along the epidermis (Supplementary Fig. S8F, arrows).

### Promoter activity in stolons and tubers

Because of the functional association of *StBEL5* RNA and the process of tuberization, *StPTB1*_*prom*_ and *StPTB6*_*prom*_ expression was investigated during tuber development. Stolons and new tubers were harvested from mature soil-grown plants and separated into four categories based on their developmental stage (Fernie and Willmitzer, 2001). GUS staining suggested that *StPTB6*_*prom*_ expression was stronger in stolons than in new tubers (right column, Supplementary Fig. S9A at *JXB* online), whereas GUS staining of *StPTB1*_*prom*_ stolons and tubers was essentially the same (left column, Supplementary Fig. S9A). Manual dissection of t2 tubers revealed that both the *StPTB1*_*prom*_ and the *StPTB6*_*prom*_ were active in vascular strands of new tubers (Supplementary Fig. S9A, arrows). Quantification of the activities in these organs revealed a peak of activity for the *StPTB6*_*prom*_ in early, induced stolons that steadily decreased as tubers formed (Supplementary Fig. S9B, black bars), whereas *StPTB1*_*prom*_ activity exhibited no such developmental regulation (Supplementary Fig. S9B, grey bars).

To explain *StPTB6* activation during early tuberization, the *StPTB6* promoter sequence was screened for tuber and sucrose inductive motifs. Three putative tuber-specific sucrose-responsive (TSSR) elements present in the promoters of class I patatin and proteinase inhibitor II genes (Grierson *et al.*, 1994) were identified in the promoter of *StPTB6* but not *StPTB1* (Supplementary Fig. S9C at *JXB* online). These are designated TSSR1, TSSR2, and TSSR3 (Supplementary Fig. S9C) and in the *patatin* promoter direct tuber-abundant expression. As an example of conservation, alignment of the putative TSSR2 motif from *StPTB6* with the proximal *patatin* TSSR motif revealed nucleotide sequence matches in the B-box, the repression region, and the A-box of 59, 69, and 74%, respectively (Butler and Hannapel, 2012).

**Fig. 3.**
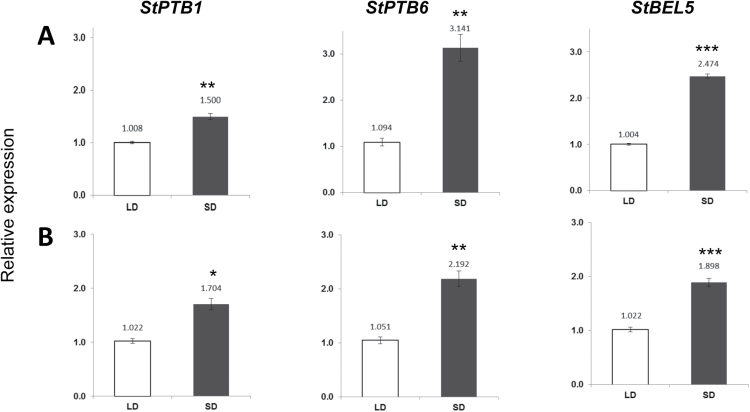
Effect of photoperiod on transcript accumulation of *StPTB1* and *StPTB6* in leaf mesophyll (A) and leaf veins (B) from the photoperiod-responsive potato species, *S. tuberosum* ssp. *andigena*. Short-day (SD) plants were harvested after 14 d of SD conditions (8h light, 16h dark). Quantitative real-time RT–PCR with gene-specific primers was used to calculate the relative amounts of RNA for each *StPTB* gene. *StBEL5* was included as a positive control. Each sample was measured in triplicate and normalized against *StActin8* RNA. The fold change in expression was calculated as the 2^−ΔΔCt^ value relative to the mean values obtained in the long-day (LD) samples. Standard errors of the means of three biological replicates are shown, with one, two, and three asterisks indicating significant differences (*P*<0.05, *P*<0.01, *P*<0.001, respectively) using a Student’s *t*-test.

*StBEL5* transcripts consistently exhibited enhanced mobility and accumulation in response to an SD photoperiod (Chen *et al.*, 2003; Banerjee *et al.*, 2006*b*). To assess expression patterns for the *StPTB* genes in response to photoperiod, RNA accumulation patterns for StPTB1 and StPTB6 were monitored in the photoperiod-sensitive *S. tuberosum* ssp. *andigena*. Under long-day (LD) conditions, there were no significant differences among the five organs tested (Supplementary Fig. S10 at *JXB* online). Under SD conditions, however, enhanced accumulation of both *StPTB1* and *StPTB6* was observed concomitantly with *StBEL5* accumulation in leaf mesophyll and leaf veins (Fig. 3). SD-induced accumulation was also observed in petioles for *StPTB1* and in lateral roots for *StPTB6* (Supplementary Fig. S11). No SD-enhanced accumulation occurred for either PTB type in stolons or stems (Supplementary Fig. S11, S12). Despite the observation that *StPTB* transcript accumulation was not induced by SDs in stems, levels of the StPTB proteins increased in both stems and stem exudate under SD conditions (Supplementary Fig. S12), suggesting some degree of photoperiod-mediated post-transcriptional regulation is in effect. StPTB protein levels were also enhanced by SDs in petioles, roots, and stolons (Supplementary Fig. S13).

### Transgenic *StPTB* plants exhibit a tuberization phenotype

**Fig. 4.**
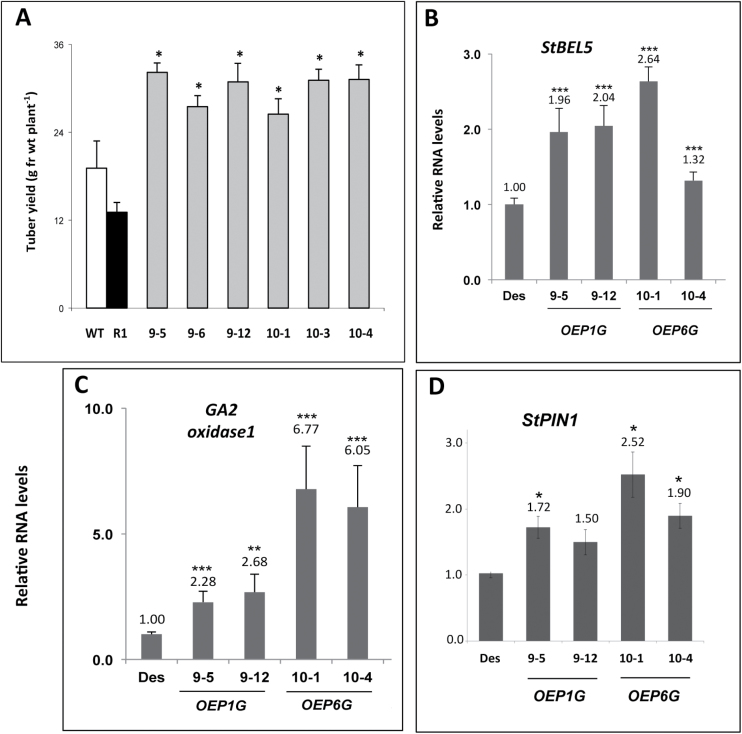
Tuber production (A) for independent Désirée transgenic overexpression lines for PTB1 (#9 lines) and PTB6 (#10 lines). Four to five replicates from select overexpression lines were grown in 13cm pots. Tuber yields (g FW per plant) were assessed at 45 d. Plants were grown under long-day conditions (16h light, 8h dark) with a fluence rate of 300 mmol m^–2^ s^–1^ at 24 °C during a 12h day cycle and 18 °C for the remaining time. Relative RNA accumulation of *StBEL5* (B), *GA2 oxidase1* (C), and *StPIN1* (D) in stolons of PTB overexpression lines after 30 d in soil. RNA was calculated using qRT–PCR and is shown in relation to wild-type (WT) levels. Des, wild-type Désirée control; R1, transgenic Désirée GFP control; OEP1G, overexpression line of StPTB1; OEP6G, overexpression line of StPTB6. Standard deviations of the means of three biological replicates are shown, with one, two, and three asterisks indicating significant differences (*P*<0.05, *P*<0.01, *P*<0.001, respectively) using a Student’s *t*-test.

To better understand the function of StPTB1 and StPTB6, ~30 transgenic overexpression (OE) lines of potato cv. Désirée were generated for both protein types, screened, and evaluated. Several high expressing lines were identified for each construct, and three to four were used in evaluating effects on RNA metabolism and phenotypes of soil-grown plants. Overall shoot fresh weight was not affected in both OE lines, but both PTB-OE types exhibited enhanced root growth (Supplementary Fig. S14 at *JXB* online). With three representative lines each, the average increase for root growth was 71% for the StPTB1-OE lines and 101% for the StPTB6-OE lines. Tuber yields from both StPTB soil-grown OE lines were more than twice that of the GFP control line, R1 (Fig. 4A). Earliness, tuber numbers, and tuber yields were also enhanced in both OE lines grown under *in vitro* conditions (Table 1).

**Table 1.** In vitro tuberization assays for StPTB1 and StPTB6 overexpression lines Explant material was from 2-week old *in vitro* plantlets grown under long-day conditions on MS medium with 2% sucrose. Eighteen axillary bud explants per line were cultured in the dark on MS medium plus 8.0% sucrose for 21 d at 25 °C. Each explant was composed of one bud and an ~0.5cm stem section. Bud explants were scored for earliness (days to first tuber), shoot or tuber formation, and total tuber yield (mg).

Transgenic line	Days to first tuber	No. of shoots	No. of tubers	Tuber yield (total mg)
Des	10	12	6	110
9-5	NA	5	13	405
9-6	6	0	18	918
9–12	7	1	17	464
10–1	6	10	8	368
10–3	6	4	14	430
10–4	7	2	16	472

Des, Désirée control. The #9 lines are StPTB1 and the #10 lines are StPTB6.

NA, data not available.

**Fig. 5.**
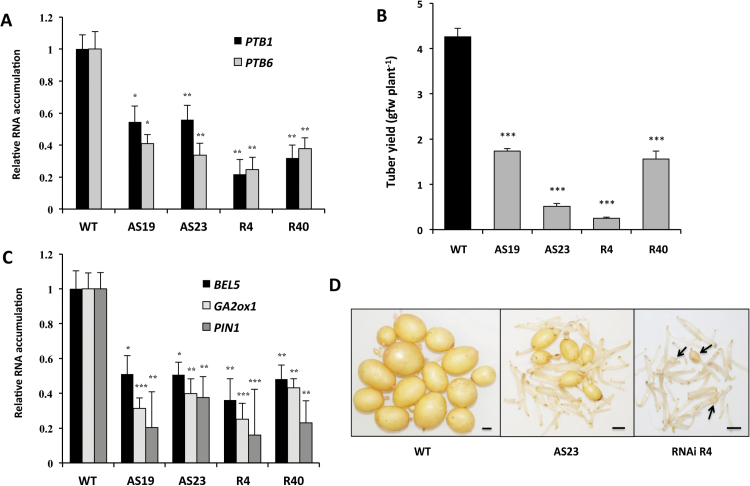
RNA suppression lines for StPTB1 and StPTB6 (A). Lines AS19 and AS23 expressed antisense sequence, whereas lines R4 and R40 expressed an RNAi sequence. Both types suppressed expression of both PTB types from stolons of plants after 15 d in soil (A). Four to five replicates from select RNAi lines were grown in 13cm pots. Tuber yields (g FW per plant) were assessed at 30 d (B and D). The tubers depicted in (D) are the pooled harvest of five plants. Arrows in (D) indicate the small tubers harvested from line R4 at 30 d. Plants were grown under long-day conditions (16h light, 8h dark) with a fluence rate of 300 mmol m^–2^ s^–1^ at 24 °C during a 12h day cycle and 18 °C for the remaining time. RNA accumulation for *StBEL5*, *StGA2ox1*, and *StPIN1* was assessed in the same stolons from suppression lines after 15 d in soil (C). RNA was calculated using qRT–PCR and is shown in relation to wild-type (WT) levels. WT, non-trangenic Désirée control. Standard errors of the means of three biological replicates are shown, with one, two, and three asterisks indicating significant differences (*P*<0.05, *P*<0.01, *P*<0.001, respectively) using a Student’s *t*-test. The size bars in (D) are equivalent to 0.5cm.

In both StPTB1- and StPTB6-OE lines, *StBEL5*, G*A2ox1*, and *StPIN1* transcript levels were enhanced in stolon tips (Fig. 4B–D). Levels of *GA2ox1* in StPTB6-OE lines increased by >6-fold. *GA2ox1* is an important marker gene involved in reducing gibberellin levels during the onset of tuber formation (Kloosterman *et al.*, 2007) and is induced by StBEL5 through an interaction with tandem TTGAC elements present in the promoter of *GA2ox1* (Lin *et al.*, 2013). *StPIN1* is also a tuberization marker and was previously confirmed to be induced by StBEL5 (Navarro *et al.*, 2011; Hannapel *et al.*, 2013). To complement the OE results, ~30 transgenic suppression lines were generated and screened for reduced *StPTB* RNA levels. The design of the antisense/RNAi sequence provided suppression of RNA accumulation for both StPTB types (Fig. 5A). Based on *StPTB* levels, four lines were selected and evaluated for tuber yield and *StBEL5* RNA levels. All four suppression lines exhibited a reduction in tuber yields and *StBEL5* RNA levels (Fig. 5B–D). Consistent with a reduction in StBEL5 activity, two known targets of StBEL5, *StGA2ox1* and *StPIN1*, also exhibited a decrease in transcript levels (Fig. 5C). Highlighting the specificity of StPTB activity in tuber development, yields were reduced by 8- and 16-fold for AS23 and R4, respectively (Fig. 5B, D), whereas shoot fresh weight among these lines exhibited no difference relative to the WT (Supplementary Fig. S15 at *JXB* online).

### The StPTB proteins regulate stability and movement of *StBEL5* RNA

**Fig. 6.**
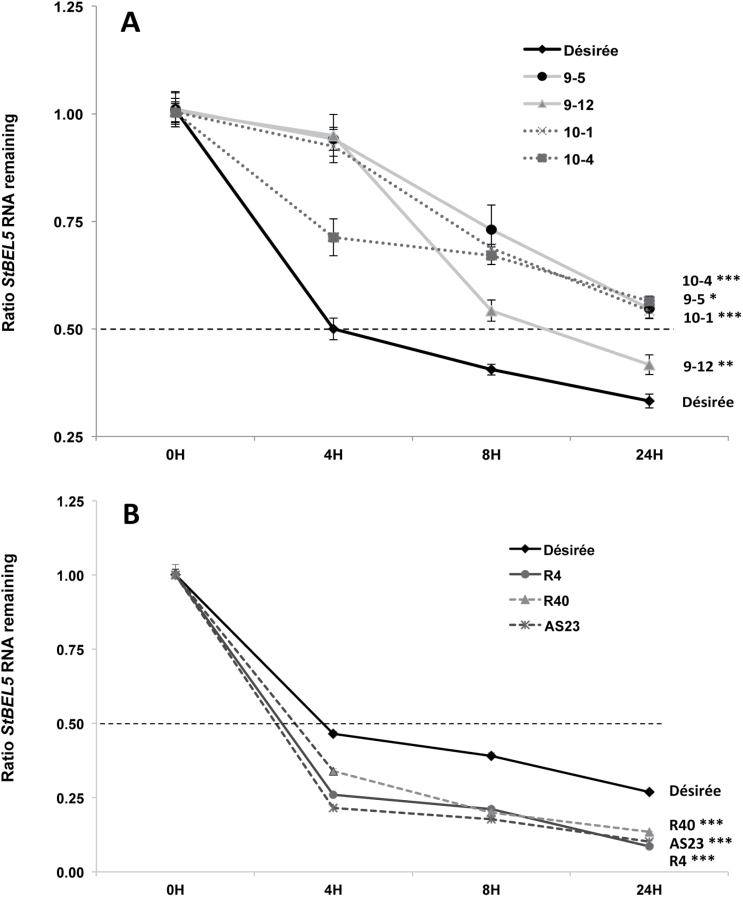
Stability of *StBEL5* transcripts in wild-type Désirée (black solid line, A and B) and overexpression lines (A) of StPTB1 (lines 9-5 and 9–12, grey, solid lines) and StPTB6 (lines 10–1 and 10–4, dotted lines) or the three StPTB1/6 suppression lines, R40, AS23, and R4 (B). Shoot tips (1cm) from *in vitro* cultured plants grown under long days were harvested and incubated in a 250ml flask with incubation buffer (Seeley *et al.*, 1992) on a shaker at 75rpm for 30min. Cordycepin was added to a final concentration of 0.6mM and the flask containing the samples was vacuum infiltrated for 45 s. Five to six shoot tips were harvested and pooled at each time interval, blotted dry, and frozen immediately in liquid nitrogen. RNA was extracted and quantitative real-time RT–PCR with gene-specific primers was used to calculate the relative amounts of *StBEL5* RNA. Each sample was measured and normalized against *StActin8* RNA. The ratio of RNA remaining for each sample was calculated as the 2^−ΔΔCt^ value relative to the mean values obtained from samples at 0h. The straight dashed line indicates the half-life point. *StActin8* RNA degraded at the same rate for all lines tested here. Standard errors of the means of two biological and two technical replicates are shown. One, two, and three asterisks indicate significant differences in the rate of degradation relative to the wild-type control (*P*<0.05, *P*<0.01, *P*<0.001, respectively) using regression analysis.

To gain insight into the mechanism of the StPTB protein interactions with *StBEL5* RNA, an RNA stability assay was performed using *in vitro* grown shoot tips of the overexpression lines and wild-type Désirée. RNA stability of *StBEL5* was enhanced in both StPTB-OE types. Significant differences were observed for the rate of *StBEL5* degradation in all four StPTB-OE lines (Fig. 6A). In wild-type plants, *StBEL5* reached a half-life as early as 4h, whereas, even after 8h, with a degradation ratio of 0.40 in wild-type plants, *StBEL5* transcripts had not reached half-life in any of the StPTB-OE lines, with an overall average ratio of 0.66. After 24h, only one of the four StPTB-OE lines had dropped below the half-life for *StBEL5* RNA (Fig. 6A). These enhanced stability rates for *StBEL5* transcripts were observed in two separate experiments. In contrast, the stability of *StBEL5* decreased significantly in the StPTB suppression lines relative to the wild-type control (Fig. 6B). After 24h, relative amounts of *StBEL5* were at 0.27 for the wild type, whereas the suppression lines exhibited levels of 0.13 or lower. These results, coupled with the binding assays from Fig. 1, strongly suggest that StPTB1 and StPTB6 contribute to the overall stability of *StBEL5* RNA *in planta* through a direct protein–RNA interaction and very probably contribute to the high abundance levels of *StBEL5* transcripts observed throughout the potato plant (Chen *et al.*, 2003; Sharma *et al.*, 2014).

**Fig. 7.**
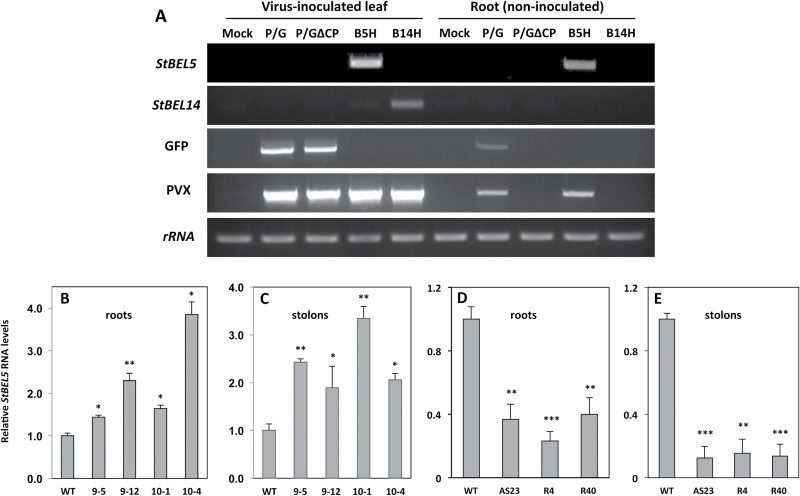
Analysis of RNA movement using a PVX vector system in wild-type (WT) Désirée (A). PVX-based *StBEL5* RNA movement into roots (B and D) and stolons (C and E) of StPTB overexpression (B and C) and suppression (D and E) lines, 8 d post-inoculation. This whole-plant system provides an accurate assessment of long-distance RNA movement (Li *et al.*, 2009). After inoculation in leaves, the *PVX* viral RNA cannot traffic long distance without its coat protein (A; root, non-inoculated P/G∆CP lane). When full-length *StBEL5* RNA replaced the coat protein sequence, transport of *StBEL5* RNA from inoculated leaves into non-inoculated roots of WT Désirée plants was confirmed (A; root, non-inoculated B5H lane). Both the non-mobile *StBEL14* RNA and GFP without a coat protein fusion were unable to transport long distance (A; root, non-inoculated P/G∆CP lane). For quantification, RNA was extracted and quantitative real-time RT–PCR with gene-specific primers (Supplementary Table S5 at *JXB* online) was used to calculate the relative amounts of *StBEL5* RNA that trafficked into roots (B and D) or stolons (C and E). Each sample was measured and normalized against *StActin8* RNA. RNA values were calculated as the 2^−ΔΔCt^ value relative to the mean values obtained from WT samples. StPTB1- and StPTB6-OE lines are designated #9 and #10, respectively. The three suppression lines are designated AS23, R4, and R40. Standard deviations of the means of two biological replicates with two technical replicates are shown, with one, two, and three asterisks indicating significant differences (*P*<0.05, *P*<0.01, *P*<0.001, respectively) using a Student’s *t*-test. For (A), Mock, inoculation with water; P/G, PVX vector with coat protein and GFP (mobile); P/G∆CP, PVX vector with GFP but no coat protein (non-mobile); B5H, PVX vector with full-length *StBEL5* plus histidine tag but no GFP or coat protein; B14H, PVX vector with full-length *StBEL14* plus histidine tag but no GFP or coat protein.

**Fig. 8.**
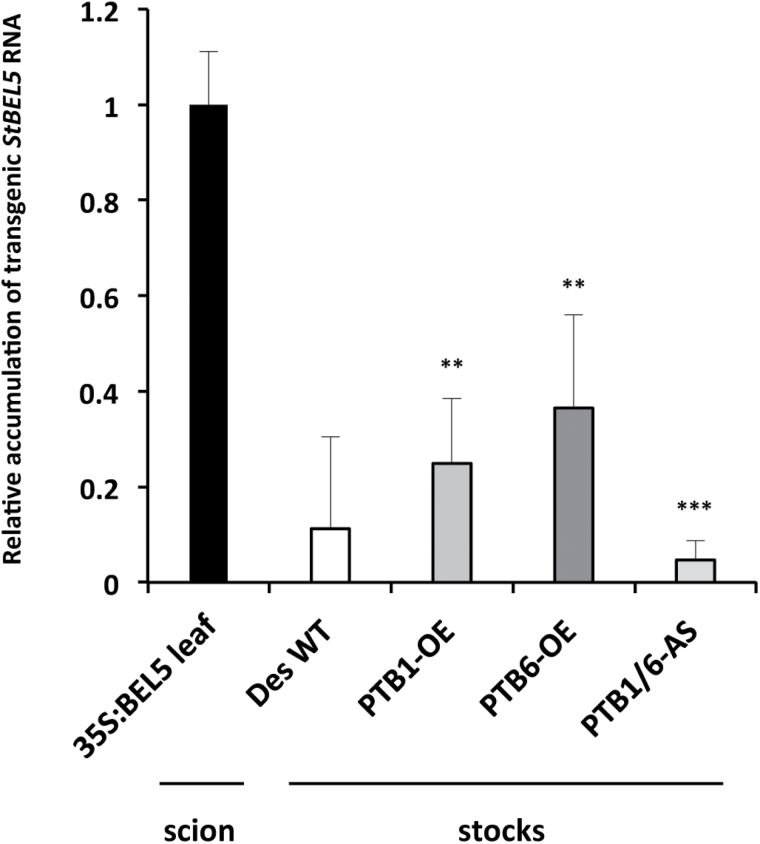
Movement of transgenic *StBEL5* RNA from the 35S:BEL5 transgenic scion into stocks of wild-type (WT) Désirée and transgenic lines overexpressing StPTB1 (PTB1-OE line 9–12), StPTB6 (PTB6-OE line 10–1), and a line that suppresses both StPTB1 and StPTB6 (PTB1/6-AS line 23). All transgenic lines are from the potato cv. Désirée. Heterografts were performed *in vitro* and maintained in tissue culture for 3 weeks, and then moved to soil for 3 weeks before harvesting. For quantification, RNA was extracted from either leaves of the scion or stolon tips (new tubers) of the stocks, and qRT–PCR was used to calculate the relative amounts of transgenic *StBEL5* RNA that trafficked across the graft union and down into stolon tips. Each sample was measured and normalized against *StActin8* RNA. RNA values were calculated as the 2^−ΔΔCt^ value relative to the mean values obtained from leaf samples of the 35S:BEL5 scion. Standard deviations of the means of two biological replicates with two technical replicates are shown, with two and three asterisks indicating significant differences from the WT stock stolons (*P*<0.01 and *P*<0.001, respectively) using a Student’s *t*-test.

To assess the effect of these StPTBs on *StBEL5* movement, RNA movement assays were performed utilizing a PVX vector system (Li *et al.*, 2009). This whole-plant system has proven to be accurate and specific in the assessment of RNA movement over a short period of time (Li *et al.*, 2009, 2011). As presented here (Fig. 7), by utilizing RNA-specific primers and real-time qRT–PCR, this system is especially useful for the reproducible quantification of RNA transport. After inoculation in leaves, the *PVX* viral RNA cannot traffic long distance without its coat protein. When full-length *StBEL5* RNA replaced the coat protein sequence, transport of *StBEL5* RNA from inoculated leaves into non-inoculated roots of wild-type Désirée plants was confirmed (Fig. 7A). Both the non-mobile *StBEL14* RNA and GFP without a coat protein fusion were unable to transport long distance (Fig. 7A). In the StPTB-OE lines, long-distance transport of full-length *StBEL5* RNA (minus the PVX coat protein) to both roots and stolons was enhanced relative to movement in wild-type plants (Fig. 7B, C). In comparison, again using the PVX system, suppression lines of StPTB1/6 repressed *StBEL5* transport to both roots and stolons (Fig. 7D, E). *StBEL5* movement into stolons was decreased by as much as 8-fold in suppression line AS23. To verify movement in a second system, heterografts were made *in vitro* with CaMV 35S:BEL5 transgenic scions grafted onto wild-type, StPTB-OE, or StPTB suppression stocks (Fig. 8). After 21 d in soil, newly formed tubers were harvested and the amount of transgenic *StBEL5* RNA was measured. Consistent with the results from the PVX system, *StBEL5* RNA accumulation was positively correlated with the level of the StPTB proteins (Fig. 8). In these experiments, the source (scion) of the mobile transgenic *StBEL5* RNA is the same for all stocks analysed. Overall, these results suggest that steady-state levels of *StBEL5* in both stolon tips and roots are due to the stability and movement of its mRNA both mediated by an interaction with StPTB proteins.

## Discussion

### RNA binding function of StPTB1 and StPTB6

Among the six PTB proteins of potato, StPTB1 and StPTB2 are most similar in sequence to the phloem-mobile PTB protein of pumpkin, CmRBP50 (Ham *et al.*, 2009), and to AtPTB3 of *Arabidopsis* (Rühl *et al.*, 2012). CmRBP50 binds specifically to polypyrimidine tract motifs of CUCU or UCUU present in the sequence of the phloem-mobile RNA, *CmGAI* (Ham *et al.*, 2009). In animals, the PTB protein contains four RRMs that bind to RNA sequence containing four separate CU motifs determined by the spatial arrangement of the β-sheet structures and RRM linkers of the protein (Oberstrass *et al.*, 2005; Auweter and Allain, 2008; Sawicka *et al.*, 2008; Clerte and Hall, 2009). Although the four-motif model is widely accepted, a definitive, discrete PTB-binding sequence cassette has not been established (Schmid *et al.*, 2007). Ham *et al.* (2009) demonstrated binding with sequences containing two, three, or four CU motifs, but showed no relationship for any of these motifs with the capacity for movement or any effect on RNA stability. Both StPTB1 and StPTB6 bound to CU-rich sequence present in the 3′-UTR of *StGAI* and *StBEL5* (both containing five CU motifs within 140 nucleotides of sequence, Supplementary Fig. S5 at *JXB* online) but not to sequence without these motifs (Fig. 1). Previous work with *StBEL5* RNA movement and stability assays demonstrated that deletion of its 3′-UTR resulted in decreased mobility and stability of the *BEL5* transcript (Banerjee *et al.*, 2006*b*, 2009). Conversely, addition of the 3′-UTR resulted in enhanced, localized movement of another non-mobile *StBEL* RNA (Banerjee *et al.*, 2009). The functional role of StPTB1 and StPTB6 in enhancing movement and stability of *StBEL5* (Figs 6, 7) and their capacity for binding to 3′-UTR sequence of *StBEL5* (Fig. 1; Supplementary Fig. S5) strongly suggest that this interaction is pivotal for regulating *StBEL5* RNA movement. Several reports have shown that PTB proteins increase the stability of target mRNAs (Tillmar and Welsh, 2004; Pautz *et al.*, 2006; Xu and Hecht, 2007). In mouse, a PTB protein regulates the circadian oscillation of a core clock gene by controlling its mRNA degradation through an interaction with the 3′-UTR (Woo *et al.*, 2009). *StBEL5* is a very abundant phloem-mobile RNA that functions in regulating tuber formation in potato (Banerjee *et al.*, 2006*b*). It is transcribed in the phloem of petioles and leaf veins and is transported to stolon tips to activate tuberization (Banerjee *et al.*, 2006*b*). Overexpression of the StPTBs enhanced steady-state levels of *StBEL5* in stolon tips and leaf veins (data not shown) and increased tuber production, whereas suppression lines affected the opposite trend. In petioles, the promoter of *StPTB1* was active in CCs of phloem but not the SEs (Fig. 2A; Supplementary Fig. S8C, D). Similar to this pattern of expression, *RBP50* transcripts were detected only in CCs, whereas RBP50 protein was detected in both CCs and SEs (Ham *et al.*, 2009). Transcriptional activity of *StBEL5* was observed in leaf veins and petioles, and specifically in phloem parenchyma and CCs of petioles (Banerjee *et al.*, 2006*b*).

### The role of StPTB1 and StPTB6 in regulating development

The pronounced effect on RNA accumulation levels of *StPTB1* and *StPTB6* mediated by SDs (Fig. 3) may help to explain some of the post-transcriptional dynamics that control patterns of accumulation for *StBEL5*. SDs activate tuber formation, but in leaves, *StBEL5* promoter activity is activated by light with no observable effect mediated by photoperiod (Chatterjee *et al.*, 2007). *StBEL5* transcript levels in leaves and its capacity to move, however, increase under SDs (Chen *et al.*, 2003; Banerjee *et al.*, 2006*b*). Based on binding and stability assays and the temporal and spatial control of *StPTB1* and *StPTB6* expression in relation to *StBEL5* transcription, it would appear that both StPTB proteins can potentially interact with *StBEL5* to facilitate stability and transport of its mRNA. Under an inductive photoperiod, both StPTB1 and StPTB6 may function at the leaf and petiole source to escort *StBEL5* transcripts through the phloem to stolon tips and roots. Such a model is consistent with the enhanced levels of StPTB protein observed in stem exudate under SD conditions (Supplementary Fig. S12 at *JXB* online). Concordant with their role in animal systems (Besse *et al.*, 2009; Karakasiliotis *et al.*, 2010) and the repressive effect of *StBEL5* UTRs on translation (Banerjee *et al.*, 2009), these same RNA-binding proteins very probably also regulate translational control of *StBEL5*.

In this system, it is proposed that SD-enhanced expression of the potato PTB proteins leads to induced transport of *StBEL5* to underground organs and an increase in root and tuber growth. Overexpression lines of StPTB1 and StPTB6 support this model as enhanced tuberization was observed in both lines concomitant with changes in the patterns of RNA metabolism (Fig. 4). This overexpression phenotype can be explained by an increase in *StBEL5* transcripts leading to the induction of tuber identity genes. *StBEL5* levels go up in stolons where *StBEL5* is translated, leading to enhanced binding to its KNOX partner. In tandem, this complex activates transcription of *GA2ox1* (and other genes in the tuber pathway) leading to an increase in steady-state levels of its mRNA (Lin *et al.*, 2013) and a reduction in levels of bioactive gibberellins. The overexpression, accumulation, and movement of *StBEL5* RNA have been consistently correlated with increased tuber production (Chen *et al.*, 2003; Banerjee *et al.*, 2006*b*, 2009). Here it is shown that both StPTB types bind to CU-rich RNA sequence, enhance stability and movement of *StBEL5*, and significantly increase tuber and root production. Through StPTB protein–*StBEL5* interactions, *StBEL5* RNA may be protected and localized to its functional sites in stolons and roots. Despite this observed effect on tuber development for the transgenic StPTB lines, shoot growth was essentially unchanged (Supplementary Figs S14C, D, S15A at *JXB* online).

PTB proteins have been observed to function in a diverse array of processes associated with RNA metabolism (Kuwahata *et al.*, 2007; Xu and Hecht, 2007; Ham *et al.*, 2009; Karakasiliotis *et al.*, 2010; Rühl *et al.*, 2012). The results presented here are consistent with the premise that PTB proteins of plants play an important role in escorting full-length mRNAs through the SE system. Despite significant research on the biochemical properties of CmRBP50, however, very little is known about how it functions in regulating development. This report presents novel results linking expression of two PTB proteins of potato to a specific phenotype, tuber formation. The mechanism of this effect on tuberization is strongly correlated to localization and stability of the mobile signal, *StBEL5*, and to the activity of its transcriptional targets. The use of transgenic plants in this study illuminates the function of StPTB proteins and provides an enlightening tool for understanding how they regulate a specific phase of plant growth. The phloem-associated promoter activity of *StPTB1* and *StPTB6*, the regulated activity during early stages of tuberization, the binding affinity of both StPTB proteins for the 3′-UTR of *StBEL5*, and the dramatic tuberization phenotypes of the StPTB overexpression and suppression lines unequivocally support the premise that the PTB1 and PTB6 proteins of potato function prominently in post-transcriptional events associated with signalling the onset of storage organ formation.

## Supplementary data

Supplementary data are available at *JXB* online.

Figure S1. Sequence alignment of polypyrimidine tract-binding proteins of potato.

Figure S2. Polypyrimidine tract-binding proteins of potato.

Figure S3. Phylogenetic analysis of polypyrimidine tract-binding proteins of plants.

Figure S4. Yeast three-hybrid analysis

Figure S5. RNA bait sequences used for the yeast three-hybrid and gel-shift assays.

Figure S6. Detection of purified recombinant PTB proteins using anti-PTB6 antibody.

Figure S7. Identification of reactive protein bands recognized by the anti-PTB6 antibody.

Figure S8. StPTB promoter activity in stems.

Figure S9. StPTB promoter activity in new tubers.

Figure S10. Expression profile of StPTB family members.

Figure S11. Effect of photoperiod on transcript accumulation of *StPTB1* and *StPTB6*.

Figure S12. Immunodetection of StPTB proteins in stem exudate.

Figure S13. Immunodetection of PTB proteins in several organs.

Figure S14. Morphological analyses of StPTB1 and StPTB6 overexpression lines.

Figure S15. Shoot and root fresh weight of RNA suppression lines for StPTB1 and StPTB6.

Table S1. Primers used for RACE and full-length cDNA cloning.

Table S2. Accession numbers of PTB proteins used in Supplementary Fig. S3.

Table S3. Gene-specific primers used for RT–PCR.

Table S4. Primers used for various constructs.

Table S5. Primers for quantitative RT–PCR and mobility assay (RMA).

Supplementary Data
