# Supplementary material for: Polypyrimidine tract-binding proteins of potato mediate tuberization through an interaction with StBEL5 RNA
Source: J Exp Bot. 2015 Aug 17;66(21):6835–47. doi: 10.1093/jxb/erv389 (PMC4623692; doi:10.1093/jxb/erv389)
Supplement: Supplementary Data [file supp_66_21_6835__index.html]

Polypyrimidine tract-binding proteins of potato mediate tuberization through an interaction with StBEL5 RNA — Polypyrimidine tract-binding proteins of potato mediate tuberization through an interaction with StBEL5 RNA — Supplementary Data 

# Polypyrimidine tract-binding proteins of potato mediate tuberization through an interaction with *StBEL5* RNA

## Supplementary Data

Data files

- Supplementary Data - Supplementary Data
