# Supplementary material for: Polypyrimidine tract-binding proteins of potato mediate tuberization through an interaction with StBEL5 RNA
Source: J Exp Bot. 2015 Aug 17;66(21):6835–47. doi: 10.1093/jxb/erv389 (PMC4623692; doi:10.1093/jxb/erv389)
Supplement: Supplementary Data [file supp_erv389_jexbot151878_file001.pdf]

Polypyrimidine tract-binding proteins of potato mediate tuberization through an interaction with StBEL5 RNA

Sung Ki Cho, Pooja Sharma, Nathaniel M. Butler, Il-Ho Kang, Shweta Shah, A. Gururaj Rao, and David J. Hannapel

Supplemental Files

Fig. S1. Amino-acid sequence alignment of polypyrimidine tract-binding (PTB) proteins of potato (*Solanum tuberosum* L.). RNA recognition motifs (RRM) are in bold. RNP1 and 2 for each RRM are underlined. CmRBP50 was identified as the core protein of a phloem-mobile RNA/protein complex in pumpkin (Ham et al., 2009).

```
StPTB6          M-----------TEPSKVIHVRNVGQEISENDLLQLFQPFGVI--TKLVMLRAKNQALLQ
StPTB1          M-----------SDPSKVVHVRNVGHEISENDLLQLFQPFGVI--TKLVMLRAKNQALLQ
StPTB7          MSTSGQQQFRYTQTPSKVLHLRNLPWDCSDEELVELCKPFGKIVNTKCNVGANRNQAFVE
CmRBP50         M-----------TEPSKVIHVRNVGHEISENDLLQLFQPFGVI--TKLVMLRAKNQALIQ

StPTB6          MQDIAAAVNAMQFYSNV-QP-SIRGRSVYVQFSSHQELTTVDQNAQGRGDEPNRILLVSI
StPTB1          MQDVPSAVKALQFYSNV-QP-SIRGRNVYVQFSSHQELTTMDQNAQGRGDEPNRILLVTI
StPTB7          FADLNQAINMVSYYASSSEPAQVRGKTVYIQYSNRNEIV----NNKSPGDVPGNVLLVTI
CmRBP50         MQDVPSAVNALQFFANV-QP-SIRGRNVYVQFSSHQELTTADQNAQGRGDEPNRILLVTI

StPTB6          HHVL-YPITVEVLHQVFSPHGIVEKIVTFQKSAGFQALIQYELTQTAISARNSLQGRNIY
StPTB1          HHML-YPITVDVLHQVFSPHGFVEKIVTFQKSAGFQALIQYQVQQSSVSARNSLQGRNIY
StPTB7          EGVEAGDVSIDVIHLVFSAFGFVQKIATFEKAAGFQALIQFSDVGTASAAREALDGRSIP
CmRBP50         HHML-YPITVEVLHQVFFPHGFVEKIVTFQKSAGFQALIQYQTRQCAISARTALQGRNIY

StPTB6          -------DGCCQLDIQFSNLDELQVSYNNERPRDFTNPNLPSEPKGK-SPQQ-GYGDAGA
StPTB1          -------DGCCQLDIQFSNLDELQVNYNNERSRDYTNPNLPSEQKGK-SSQQ-GYGD---
StPTB7          KYLLPEHVNHCHLHISYSAHTDLNIKFQSHRSRDYTNPYLPVNPTAMEGVLQPVVGPDGK
CmRBP50         -------DGCCQLDIQFSNLDELQVNYNNERSRDFTNPSLPSEPKGR-PSQQPGYGDAGG

StPTB6          MYPWQGSGPRGVGFPQMGNAAAIATAFPSGLPPGISGTNDRCTIIVSNLNP--DRIDEDK
StPTB1          MYSFQGSGAHPGGFPQMGNAEAIAAAFAGGLPPGISGTNDRCTILVSNLNS--DRINEDK
StPTB7          KK------------------------------------EPESNVLFASLENMQYAVTVDV
CmRBP50         MYALQASGAGPVGFPQMANAAAVAAAFGGGLPPGVSGTNDRCTVLVSNLNP--DRIDEDK

StPTB6          LFNLFSIYGNIVRIKHL-RNKPDHALVQMGDGFQAELAVHFLKGAMLFG---QRLEVNYS
StPTB1          LFNLCSLYGNIVSIKIL-RNKPDHALVQLGDGFQAELAVHFLKGAMLFE---KRLEVNFS
StPTB7          LNTVFSAFGTVQKIAIFEKNGQTQALIQYPDVTIAAAAKDALEGHCIYDGGYCKLHLSYS
CmRBP50         LFNLFSIYGNIARIKLL-RNKPDHALVQMGDGFQAELAVHFLKGAMLFG---KRLEVNFS

StPTB6          KYPNINTGPETRDYSNSNLNRFNRNAAKNYRYCCSPTKMIHVSSLPQDVTEEEIVAHLEE
StPTB1          KYPNITTGPDTHDYSNSNLNRFNRNAAKNYRYCCSPTKMIHLSSLPQDVTEAEIIAHLEE
StPTB7          RHTDLNVQAYSD---------------KSRDYTVPESSLLAMQQAS-AVHATPPVWHNPQ
CmRBP50         KHPNITQGADTHEYANSNLNRFNRNAAKNYRYCCSPTKMIHISSLSQEVTEEEIVNLLEE

StPTB6          HGPIVNTKLFEMNGKK--QALVLFDNEEQATEALVCQ---------------------HA
StPTB1          HGPIINSKLFEMNGKQ--QALVLFDKEEQATEALVCK---------------------NA
StPTB7          SGPVQSSAGYATTGTVPGQAPPAWNPNLQGGGSTFPSAPTGYPGHSYAPPAPAYATAVHP
CmRBP50         HGPIINTKLFEMNGKK--QALIMFDTEEQATEALVCK---------------------HA

StPTB6          T-------SLGGSIIRISFSQ------------------VQSI
StPTB1          T-------SLGSSTIRISFSQ------------------LQSI
StPTB7          PGSSQQTNHISTGSRPFSVSQ PFQPSTMPPGGVPPPGHAPYHG
CmRBP50         S-------SLSGSIIRISFSQ------------------LQSI
```

A

Name	Gene	Genome loci from blast	Identity	TC # (DFCI)
StPTB1	PGSC0003DMG400018824	PGSC0003DMT400048465	StPTB1	TC201869
StPTB6	PGSC0003DMG400023660	PGSC0003DMT400060830	StPTB6	TC202289
StPTB7	PGSC0003DMG400001427	PGSC0003DMT400003611	StPTB7	TC207584
StPTB7-1	PGSC0003DMG400009106	PGSC0003DMT400023506	TC201749	TC201749
StPTB7-2	PGSC0003DMG400002353	PGSC0003DMT400023506	homologous to PTB7 clade	BI920231
StPTB7-3	PGSC0003DMG400019613	PGSC0003DMT400050479	homologous to PTB7 clade	TC218925

B

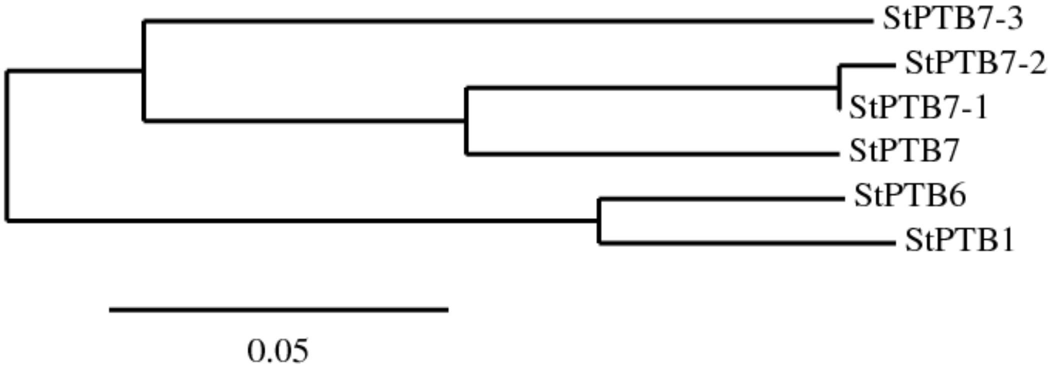

Figure S2. Classification and accession numbers of the gene family of polypyrimidine tract-binding (PTB) proteins of potato (A). Four proteins containing only three RNA-recognition motifs (RRMs) are from the PTB7 group (Supplementary Fig. S3), whereas PTB1 and -6 contain four RRMs and are similar in sequence to CmRBP50 (B) (Ham et al., 2009). As a comparison, *Arabidopsis* has one RBP50-type and two PTB7-types. Gene and Genome loci numbers are from the potato genome website (http://potatogenomics.plantbiology.msu.edu/index.html). TC accession numbers are from the DFCI Potato Gene Index (http://compbio.dfci.harvard.edu/tgi/cgi-bin/tgi/gimain.pl?gudb=potato).

Figure S3. Phylogenetic relationship of PTB proteins in plants. The deduced amino-acid sequences from the full-length cDNAs for *StPTB1*, *-6*, *-7* and TC201749 were compared to 56 PTB protein sequences. Two other StPTBs (BI920231, TC218925) were identified from the Potato Genome Consortium website. Conserved RRM domains characteristic of PTB proteins were also identified using BLAST. For comparison to other plant PTB proteins, 56 protein sequences from 15 plants (Supplementary Table S2) were analyzed along with HnRNP, human PTB, as a control. Amino-acid sequence alignments and phylogenetic analysis were performed using T-COFFEE (http://www.ch.embnet. org/software/ TCoffee.html) and graphical representation of the phylogenetic tree was performed with TreeDyn (v198.3) (Dereeper et al., 2008).

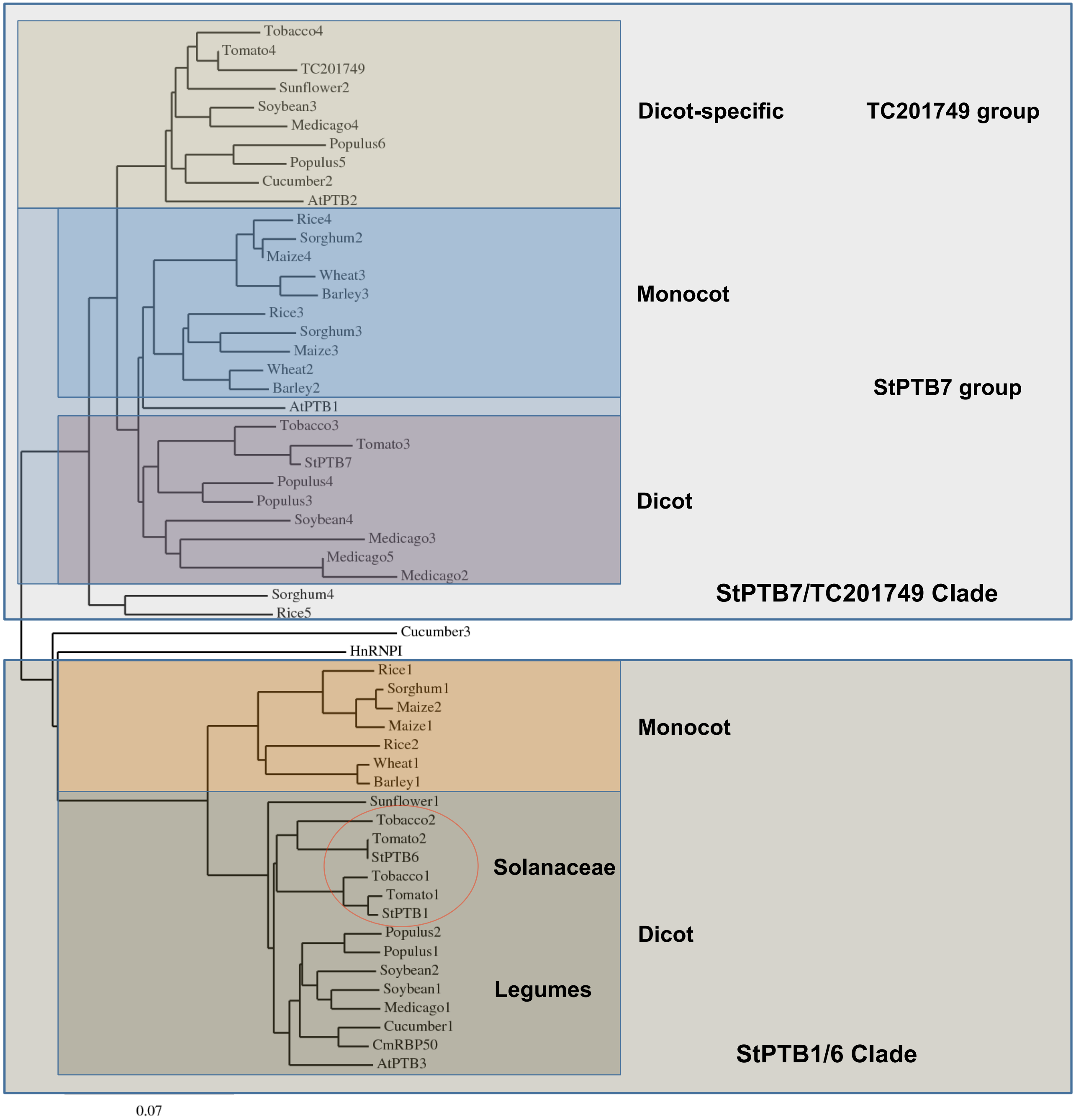

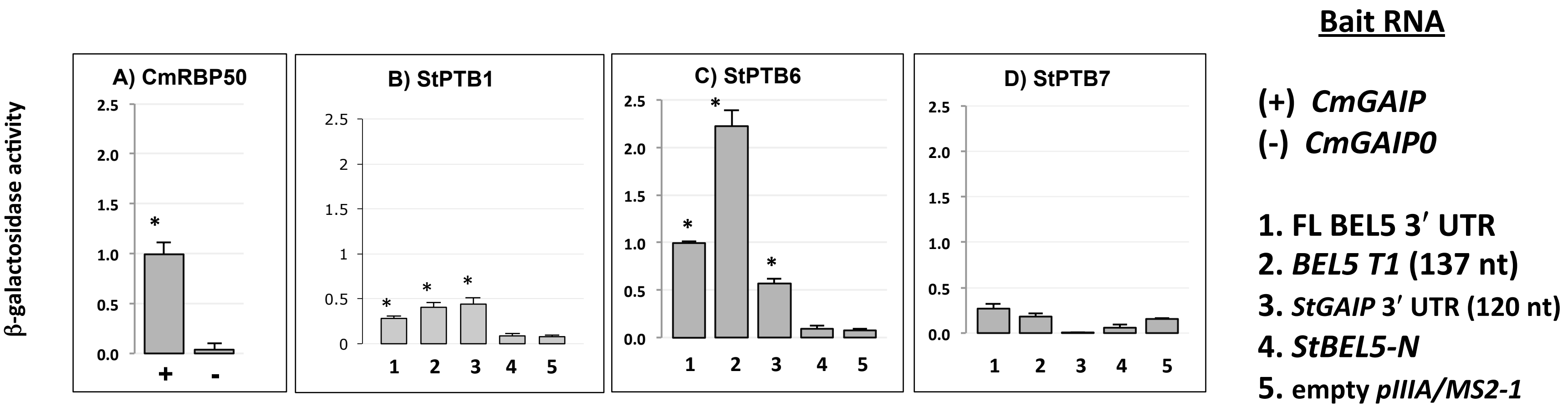

Figure S4. Yeast three-hybrid analysis. The yeast three-hybrid system (Bernstein et al., 2002) was utilized to assess the interaction of putative bait RNAs with CmRBP50, StPTB1, StPTB6, and StPTB7 proteins (A-D). Bait RNAs were known mobile RNAs: 1. *StBEL5 3′ UTR*; 2. *StBEL5 T1*; 3. *StGAIP 3′ UTR*; 4. *StBEL5*-N; 5. *pIIIA/MS2-1* empty vector. The bait RNAs tested were *CmGAIP* with CmRBP50 as a positive control, full-length *BEL5* 3′ UTR (503 nt), the *T1* fragment of the *StBEL5* 3′ UTR (137 nt), and a fragment of the 3′ UTR of *StGAI* (120 nt) with the StPTB proteins. As negative controls, *CmGAIP0* (no CU motifs, Ham et al., 2009), a cds fragment (*StBEL5-N*) of *StBEL5* (no CU motifs), and empty pIIIA/MS2-1 vector were used. *CmGAIP*, *StGAI*, and *T1* contain 6, 5, and 5 CU motifs, respectively (Supplementary Fig. S5). Based on β-galactosidase activity, the strongest interactions occurred with CmRBP50/*CmGAIP* and StPTB6/*T1* (A, C). Although, the interaction of StPTB1/full-length *BEL5 3′ UTR*, *T1* and *StGAI* were statistically significant (B), β-galactosidase activity was relatively low in these interactions compared to the strongest interactions of CmRBP50/*CmGAIP* and StPTB6/*T1*. No significant interaction of StPTB7 protein with any of the RNA baits was observed (D).

For the yeast three-hybrid constructs, the coding sequences for StPTB1, -6 and -7 were generated by PCR amplification (Supplementary Table S4-1) and introduced into *pACTII*, an activation domain vector. DNA fragments encoding various RNA baits were amplified by PCR with gene-specific primers (Supplementary Table S4-2) and cloned into the *pIIIA/MS2-1* vector to generate the hybrid RNAs. The host yeast strain YBZ-1 was utilized with RNA hybrid plasmids by the standard yeast protocol (Bernstein et al., 2002). To determine RNA-protein interaction, *HIS3* and *lacZ* were utilized as reporters. Induction of the *lacZ* reporter gene was measured with a Yeast β-galactosidase Assay Kit (Pierce Biotechnology). β-galactosidase activity was determined by following the equation; [1,000 x OD_{420}]/[time of color reaction (min) x volume of cells used (ml) x OD_{660}]. Each assay was replicated in three independent experiments. The YBZ-1 strains, plasmids *pIIIA/MS2-1* and *pACTII* were generous gifts of Dr. Marvin Wickens, University of Wisconsin, Madison.

A) *T1 sequence*

A**CUCUU**AUAUUGUGUGAGGC**CUUCU**GGCCCAAGUCGGAGGACCCAAUUUGAUACAA**CCU**
AUCAUAGGAGAAAAGAAGUGGAGACUAAAUUAAAGUAACAAAAUUUAAAGCACA**CUUU**
CUAGUAUAUAUA**CUUCUUU**

B) *StGAI* bait

UUAUAAGUCAG**CCUCUUU**AUUUA**CUUUUU**AACCA**CUUCUU**AUAUUUUG**UUCU**AGUUUA
UUUUAUUUUAUUUA**UCUU**A

C) Negative control *StBEL5 N*

UGAAGAGGUGGAGCAAAGGUACAGACAGUACCAUCACCAAAUGCAAAUAAUUGUAUUAU
CAUUUGAGCAAGUAGCAGGAAUUGGAUCAGCCAAAUCAUACA

D) 3´ UTR of *StBEL22*

UUAAGAUAGUGUA**UUC**AAACACUGCUACAUAAAUUAUGAUUUUAUAUAUAUAUAUAUU
GUCA**UCC**GAUUAGUUU

Figure S5. RNA bait sequences used for the yeast 3-hybrid and gel-shift assays (Supplementary Fig. S4; Figure 1). The *T1* sequence (A) is 137 nt in length from the 3´ UTR of StBEL5, is near the 5´ end of the UTR, and contains five cytosine/uracil motifs. The *StGAI* bait (B) is 77 nt from the 3´ UTR of *StGAI* and contains five cytosine/uracil motifs. The negative control *StBEL5 N* (C) is 101 nt from the StBEL5 cds, near the amino-terminus and contains no cytosine/uracil motifs longer than 2 nt. (D) is the 74-nt 3´ UTR of *StBEL22*. Cytosine/uracil runs of at least 3 nt are shown in bold.

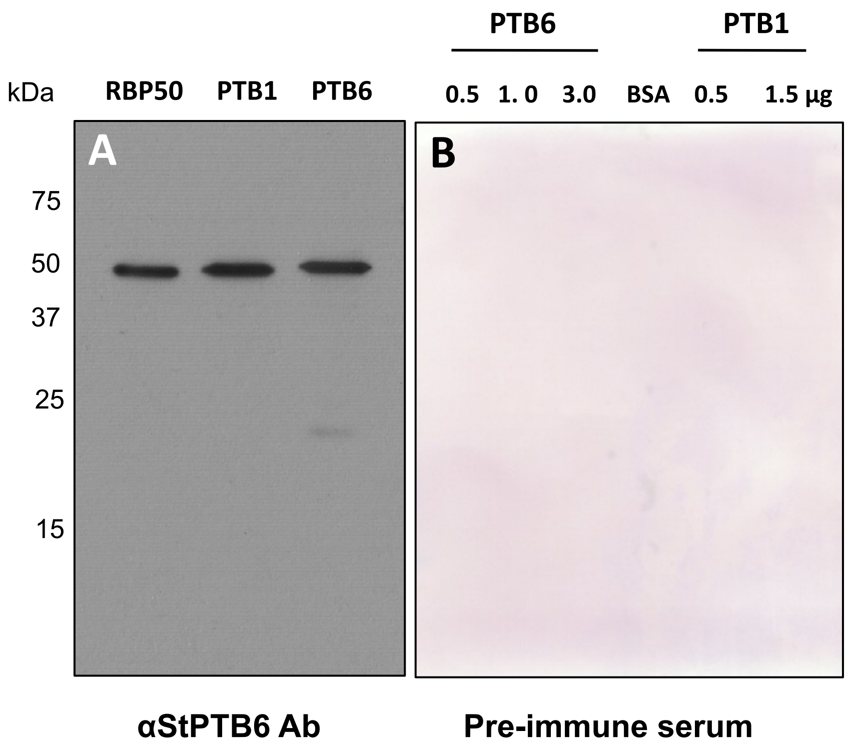

Figure S6. Detection of purified recombinant PTB proteins using anti-PTB6 antibody. One microgram of purified CmRBP50, StPTB1 or -6 protein was separated via SDS-PAGE. After blotting and incubation with antibody, all three proteins were readily detected by the anti-PTB6 antibody (A). The antibody was diluted 1:10,000 and the pre-immune serum 1:1,000 (B) for the immunodetection protocols used here.

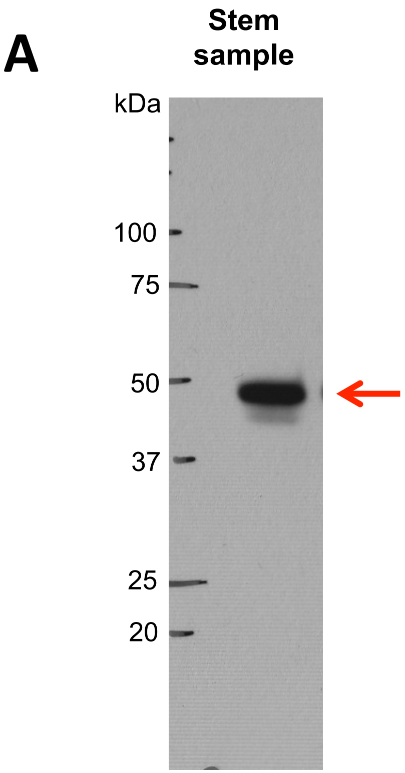

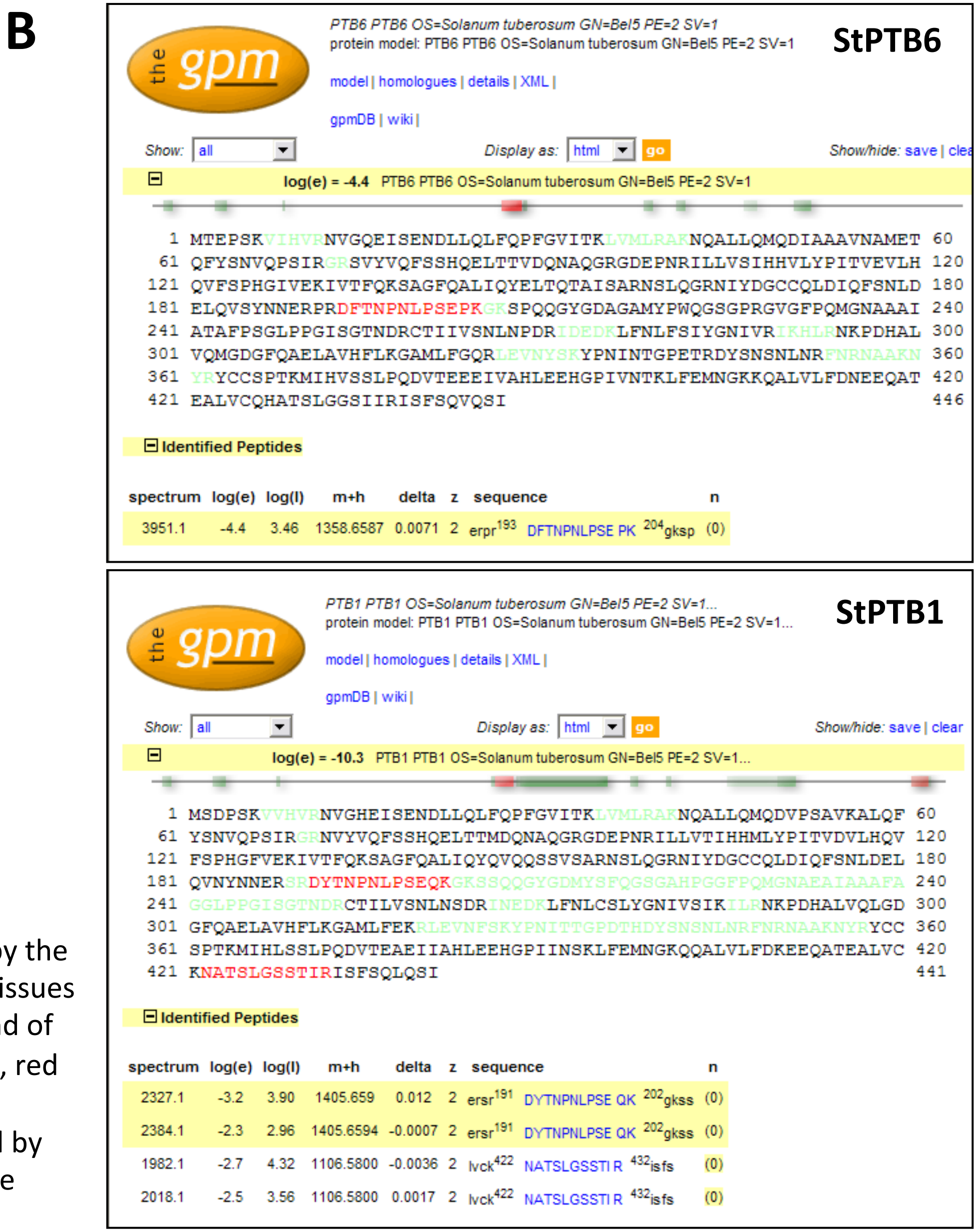

Figure S7. Identification of reactive protein bands recognized by the anti-PTB6 antibody. Total protein was extracted from various tissues of the potato cv. Désirée and separated via SDS-PAGE. The band of approximately 48 kDa recognized by the anti-PTB6 antibody (A, red arrow) was excised for analysis. Sample preparation and in-gel trypsin digestion were performed and peptides were identified by using LC-MS/MS as described previously (Shah et al., 2011). The reactive band contained both StPTB1 and -6 proteins (B).

Figure S8

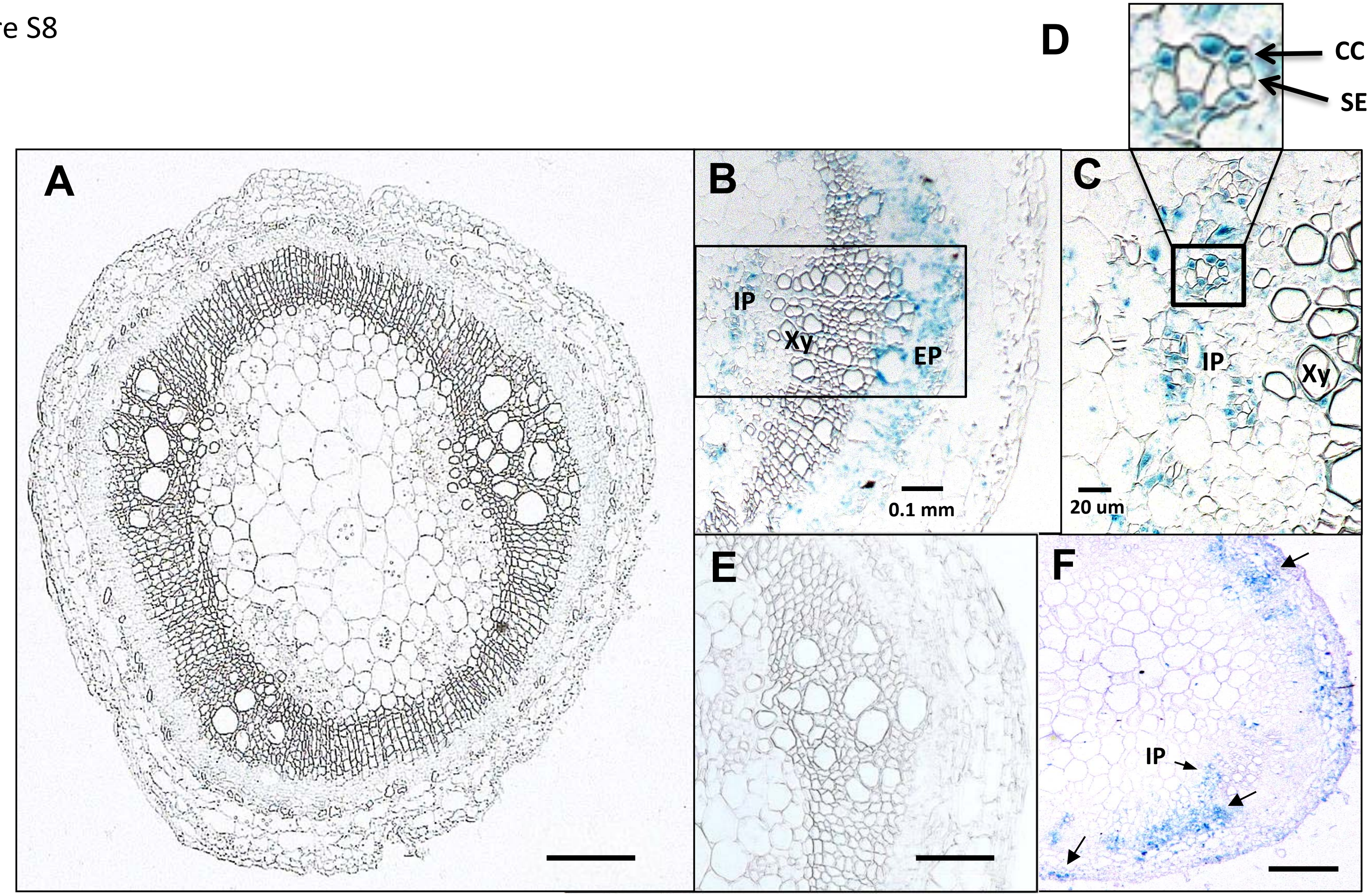

Figure S8. Localization of GUS activity within stems of *StPTB1*$_{prom}$ (A-D) and *StPTB6*$_{prom}$ (E-F) transgenic lines. Stem internodes of four-week old soil grown plants were embedded in paraffin for histochemical detection of GUS activity within tissues of the stem. Panel (A) is a transverse section of *StPTB1*$_{prom}$ with a higher magnification image of a vascular bundle (boxed area) in (B) showing xylem (Xy) and internal (IP) and external (EP) phloem tissues (C-D). Panels (E-F) are higher magnification images of a transverse section of *StPTB6*$_{prom}$ showing a vascular bundle in (E) and GUS staining in the interfascicular region, the IP, and the epidermis (F, arrows). The scale bars represent 200 μm in (A) and 100 μm in (E-F). GUS activity for the *StPTB1* promoter was detected specifically in phloem cells (B-D), both external (EP) and internal phloem (IP). Under higher magnification (D), blue staining could be detected in companion cells (CC) but not sieve elements (SE). Results presented here are representative of several sections from different stem samples.

Figure S9

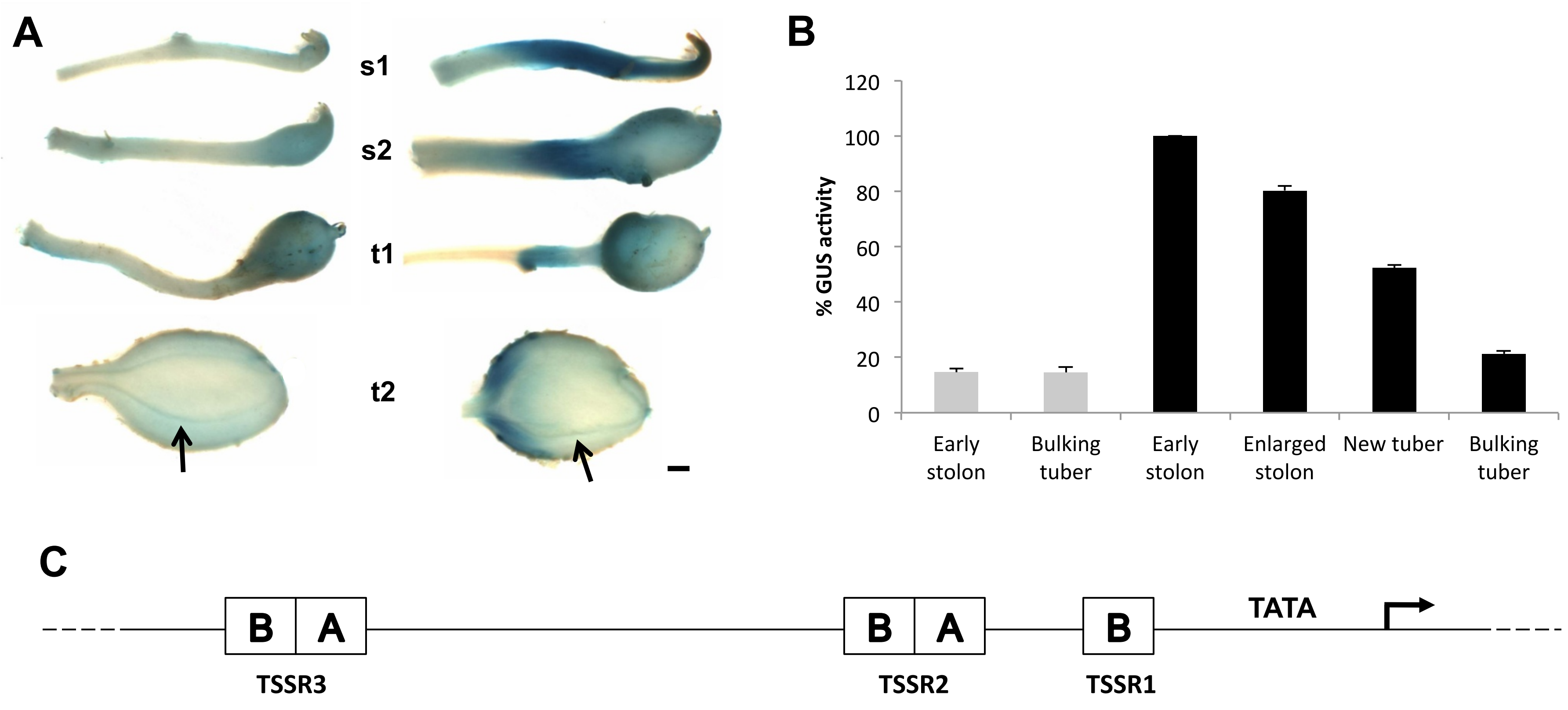

Figure S9. GUS activity within stolons and tubers of *StPTB1*$_{prom}$ (A, left column) and *StPTB6*$_{prom}$ (A, right column) transgenic lines. Stolons and tubers of various developmental stages from eight-week old soil-grown transgenic plants of *StPTB1*$_{prom}$ (B, gray bars) and *StPTB6*$_{prom}$ (B, black bars) were quantified to assess promoter activity. Two stages of stolon development designated "s1" and "s2" in panel (A) correspond to early and enlarged stolon stages, respectively. The two tuber developmental stages designated "t1" and "t2" correspond to new and bulking tuber stages, respectively. The arrows on the t2 tubers (A) indicate GUS activity in the vascular strands. Similar materials from panel A were used for quantification (B) and are shown as means of three replicate samples from four plants with standard errors. The presence of two complete and one incomplete tuber-specific sucrose responsive elements (TSSR1-3, panel C) were discovered upstream of the *StPTB6* transcriptional start site (arrow) based on *cis*-regulatory elements described in the promoters of patatin and proteinase inhibitor II genes (Butler and Hannapel, 2012). Percent GUS activity (B) was normalized to early stolon activity of the *StPTB6*$_{prom}$. The scale bar in (A) represents 1.0 mm.

Figure S10

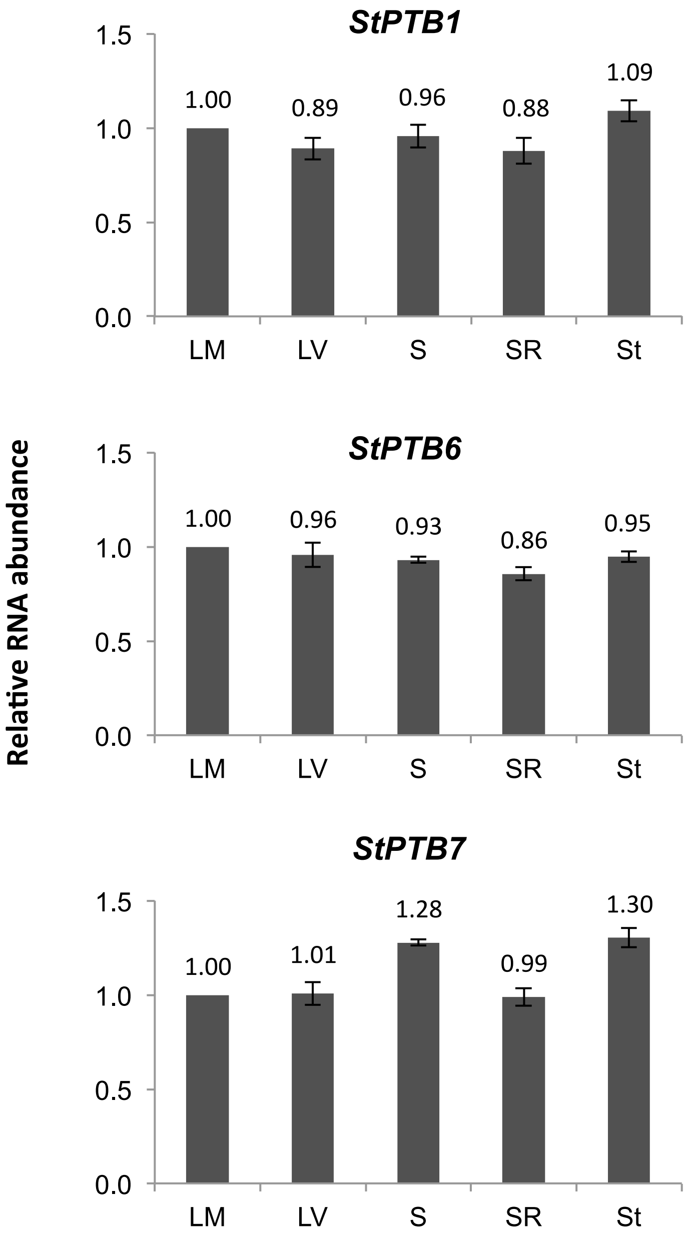

Figure S10. Relative levels of mRNAs for *StPTB1*, *-6*, and *-7* in various organs of *Solanum tuberosum* ssp. *andigena* plants grown under long-day conditions. One-step RT-PCR was performed using 150 ng of total RNA and gene-specific primers. All PCR reactions were standardized and optimized to yield product in the linear range. Homogenous PCR products were quantified by using ImageJ software (Abramoff et al., 2004) and normalized by using 18S rRNA values. Standard errors of the means of three biological replicates are shown. LM, leaf mesophyll; LV, leaf veins; S, stem; SR, secondary roots; and St, stolon tips.

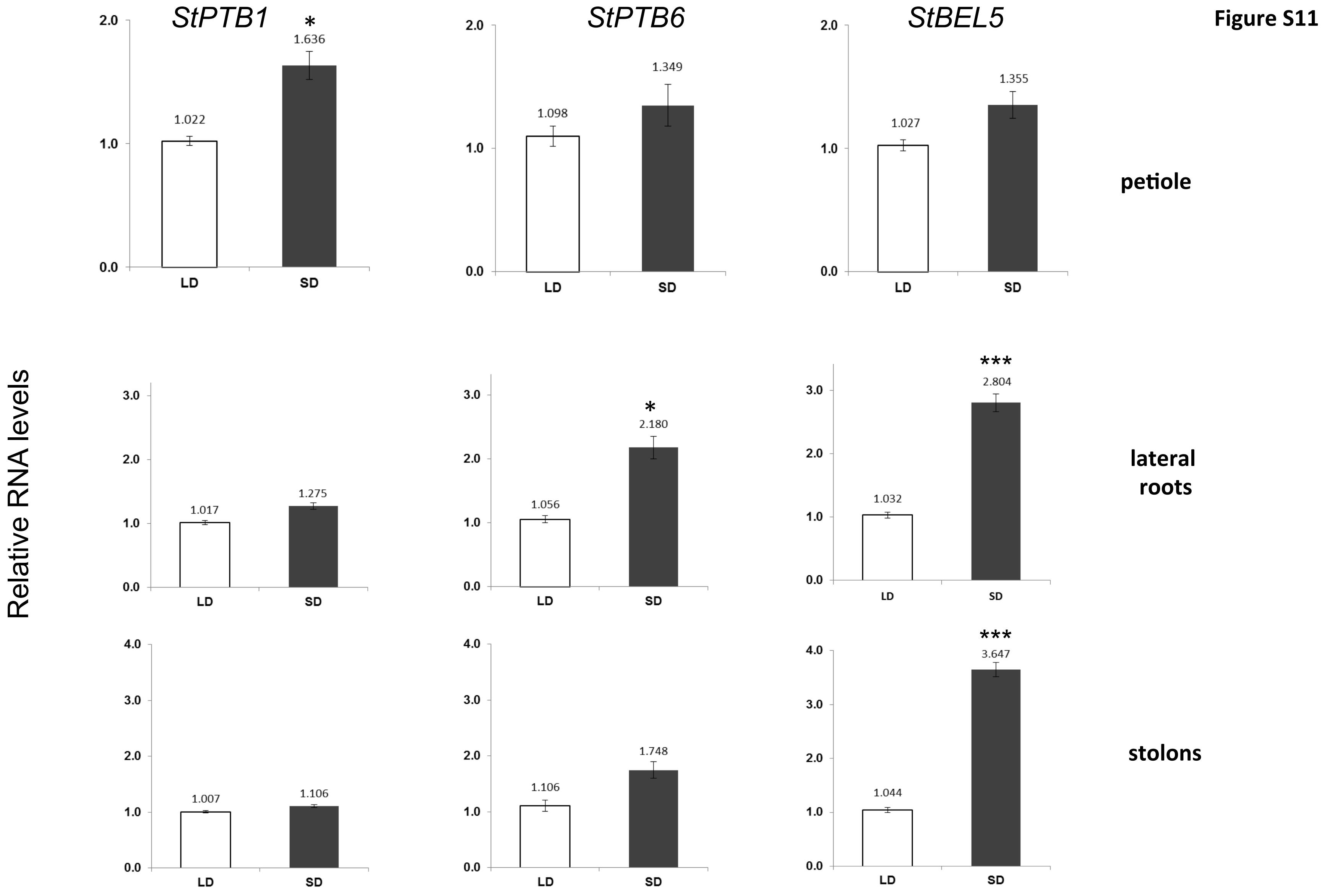

Figure S11. Effect of photoperiod on transcript accumulation of *StPTB1* and *-6* in petioles, lateral roots, and stolons from potato (*S. tuberosum* ssp. *andigena*). Short-day plants were harvested after 14d of SD conditions (8h light, 16h dark). Quantitative real-time RT-PCR with gene-specific primers was used to calculate the relative amounts of RNA for each *StPTB* gene. *StBEL5* was included as a positive control. Each sample was measured in triplicate and normalized against *StActin8* RNA. The fold change in expression was calculated as the $2^{-\Delta\Delta Ct}$ value relative to the mean values obtained in the long-day (LD) samples. Standard errors of the means of three biological replicates are shown with one asterisk indicating a significant difference ($p < 0.05$) using a Student's *t* test.

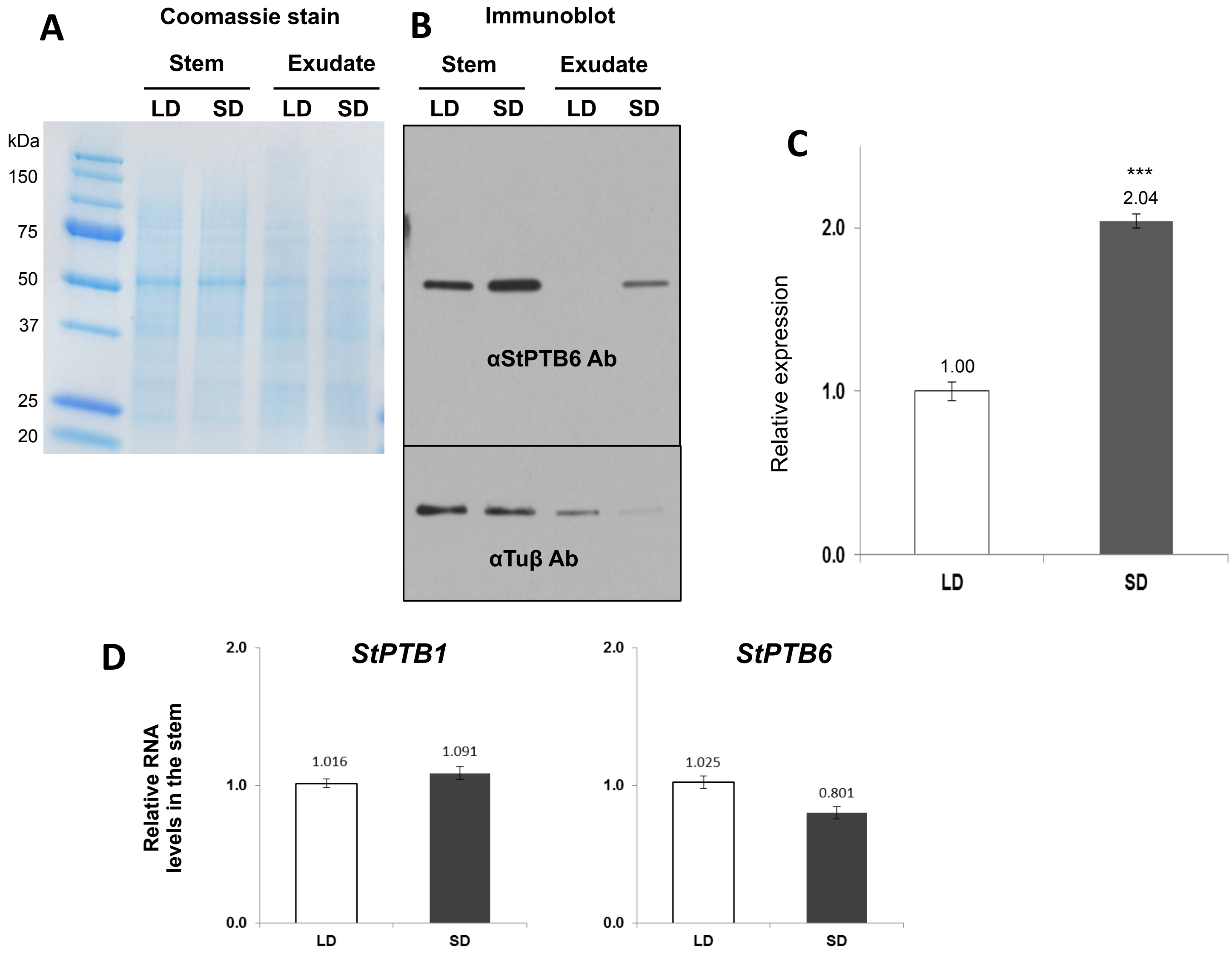

Figure S12. Immunodetection of StPTB proteins in stem and stem exudate of *S. tuberosum* ssp. *andigena* grown under long- (LD) or short-day (SD) conditions (A). Immunodetection was performed using either antibody to StPTB6 (B, upper panel) or tubulin (B, lower panel) protein. The StPTB6 antibody recognizes both StPTB1 and -6 (Figures S6-7). To collect stem exudate, stems of *Solanum tuberosum* ssp. *andigena* plants grown under long- or short-day conditions were cut into 0.5 cm pieces and centrifuged at 10,000xg for 2 min in 1.5 ml Eppendorf tubes. After spinning, the stem exudate was collected and proteins were extracted with SDS buffer (Laemmli, 1970). Forty µg of proteins from stems and exudate were separated using Any kD resolving gel (Bio-Rad), and stained using GelCode Blue Stain reagent (Thermo Scientific). The proteins were transferred to Immobilon-P transfer membranes (Millipore), stained with MemCode Reversible Protein Stain kit (Thermo Scientific) to confirm transfer efficiency, and incubated with α-StPTB6 or α-tubulin antibody (Santa Cruz). The antibody interactions were visualized using the ECL plus detection kit (GE Healthcare) and CL-XPosure (Thermo Scientific) according to the manufacturer's instructions. Signal intensities were quantified by ImageJ software (Abramoff et al., 2004), normalized with tubulin (C). Standard errors of the means of three biological replicates are shown with three asterisks indicating a significant difference ($p < 0.001$) using a Student's *t* test. RNA levels of *StPTB1* and *-6* from stems of *andigena* plants grown under LD or SD were measured using qRT-PCR (D).

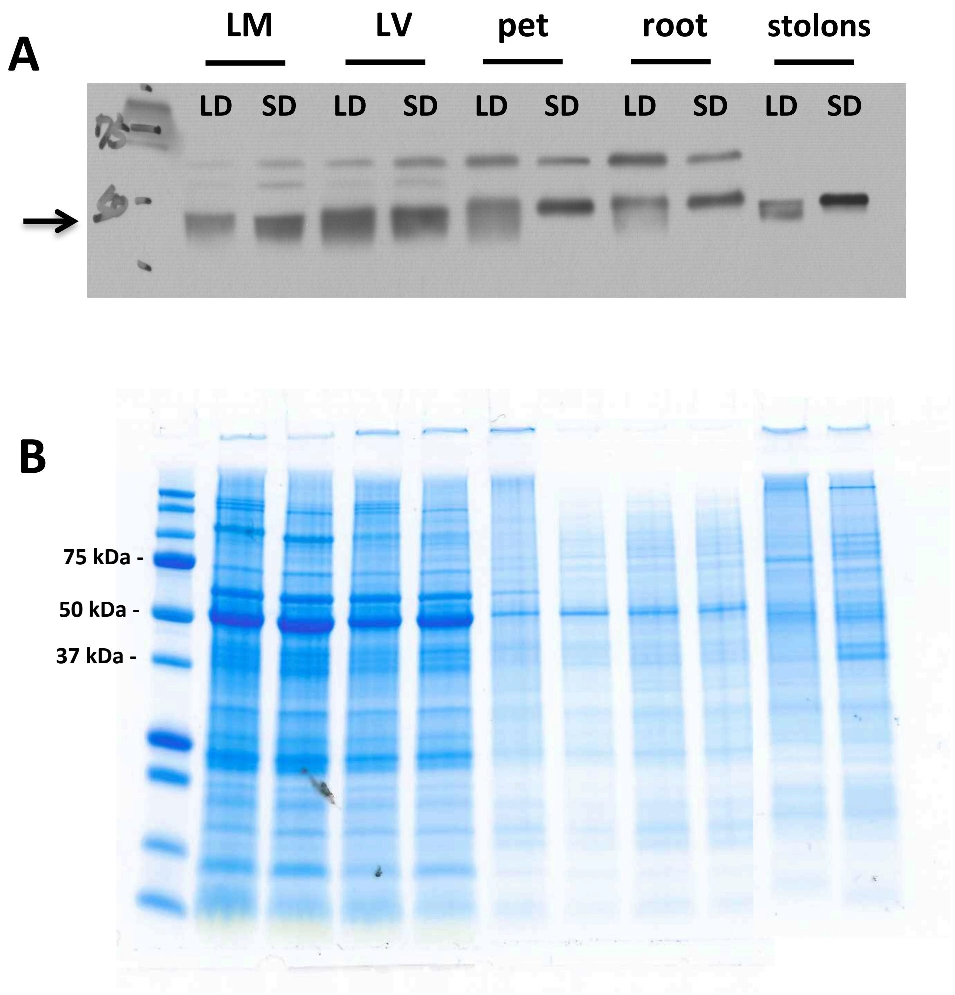

Figure S13. Immunoblot (A) of protein extracted from several organs from the photoperiod-sensitive potato, *S. tuberosum* ssp. *andigena*, grown under long-day (LD) and short-day (SD) conditions. LM, leaf mesophyll; LV, leaf vein; pet, petiole. LD plants were grown under LD for 21d. SD plants were grown under LD for 21d and then transferred to SD for 10d. The arrow in A indicates the band for the StPTB proteins at approximately 50 kDa. Coomassie blue-stained proteins and markers loaded onto the gel for transfer are shown in panel B.

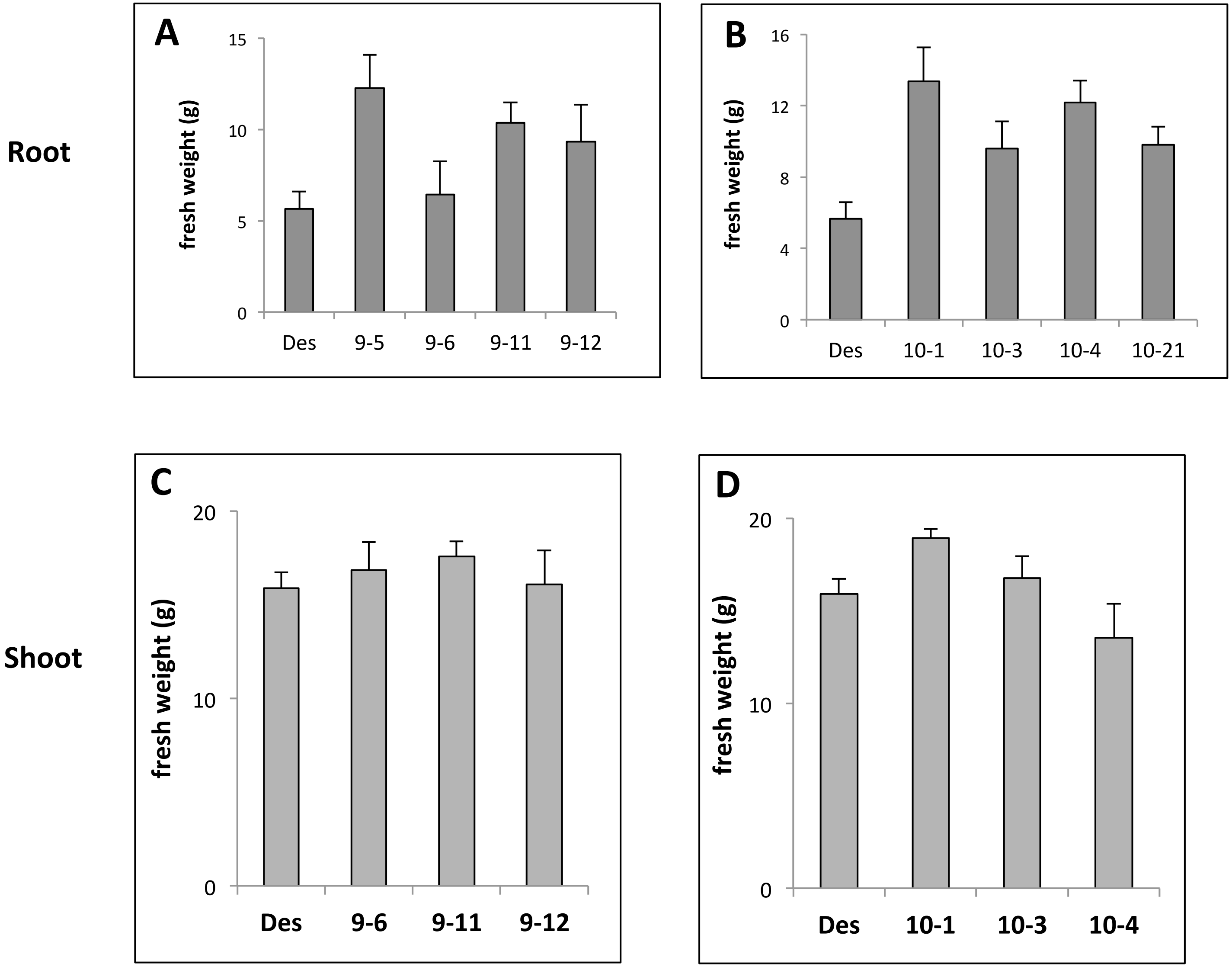

Figure S14. Morphological analyses of StPTB1 (A, C) and -6 (B, D) OE lines. Three or four replicates from selected lines were grown in vitro to a size of approximately 5 cm for two to three weeks before being transferred to soil. Plantlets were allowed to grow in 6-cm pots for two weeks and then transferred to 10-cm pots for the remainder of the experiment. Root (A-B) and shoot (C-D) fresh weight were scored from day 45 plants. All plants were grown under long-day conditions (16h light, 8 h dark) with a fluence rate of 300 µmol m^{-2} s^{-1} at 24 °C and 18 °C using a 12-h diurnal temperature cycle. Des, non-transgenic potato cv Désirée. The #9 lines are PTB1. The #10 lines are PTB6.

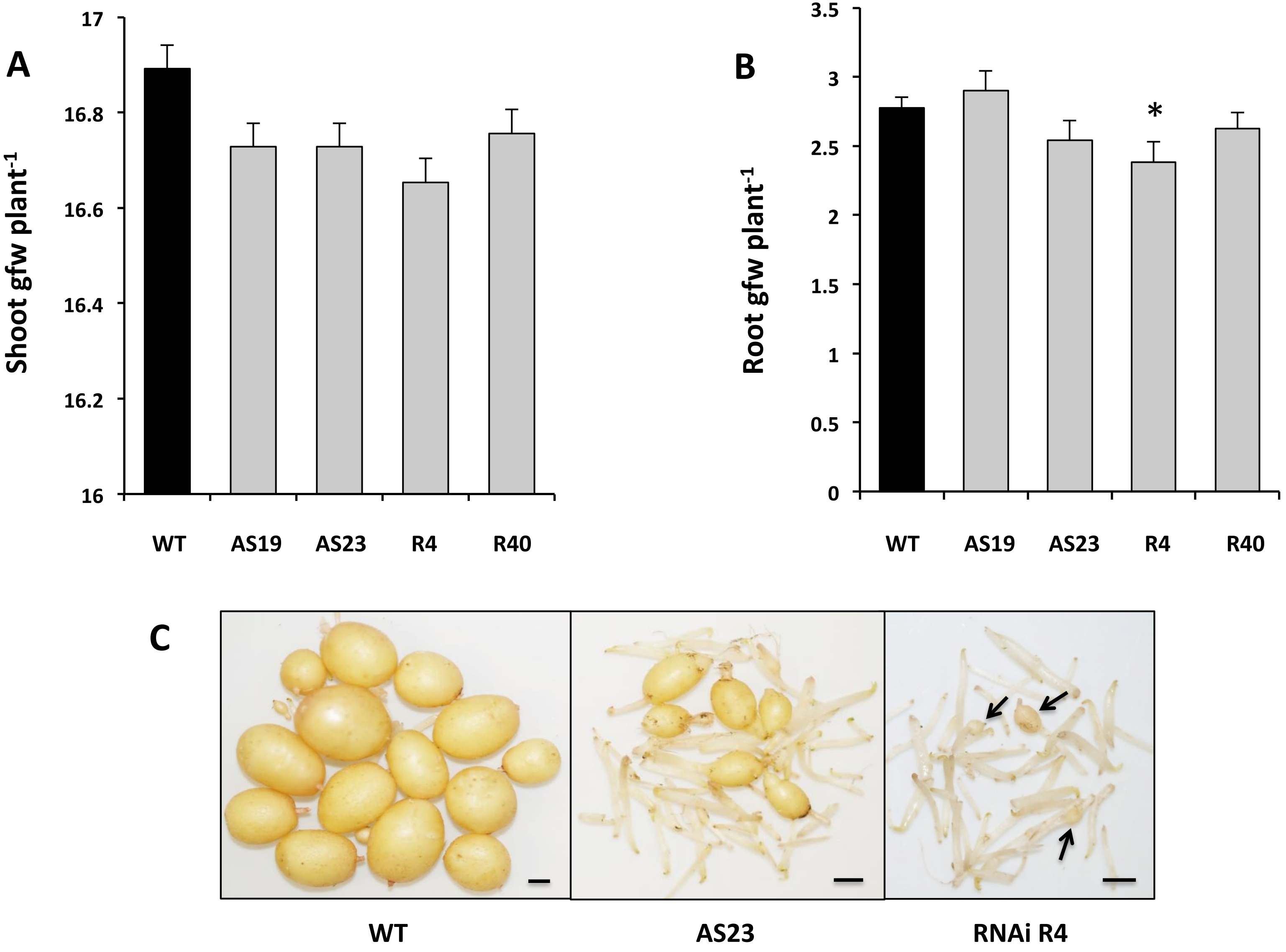

Figure S15. Shoot (A) and root (B) fresh wt of RNA suppression lines for StPTB1 and StPTB6. Lines AS19 and AS23 expressed antisense sequence, whereas lines R4 and R40 expressed a RNAi sequence. Both types suppressed expression of both PTB types. *StPTB* RNA levels in stolons for these suppression lines are shown in Fig. 5A. Plants from select RNAi lines were grown in soil in 13-cm pots. Shoot and root yields (g fr wt plant^{-1}) were assessed at 30d. Tuber yield from five plants of WT and lines AS23 and R4 harvested at 30d (C). Arrows indicate the small tubers harvested from line R4. Plants were grown under long-day conditions (16h light, 8h dark) with a fluence rate of 300 μmol m^{-2} s^{-1} at 24 °C during a 12-h day cycle and 18 °C for the remaining time. WT, non-trangenic Désirée control. Standard errors of the means of five plants are shown with the asterisk indicating a significant difference ($p < 0.05$) using a Student's *t*-test. The size bars in D are equivalent to 0.5 cm.

Table S1. Primers used for RACE, full-length cDNA and Antisense/RNAi cloning.

RACE	Outer primer (5' to 3')	Inner (nested) primer (5' to 3')
StPTB1 5' RACE	TTGAGAGGATTTGCCCTTCT	AAAGCTTGAAAACCAGCCGA
StPTB1 3' RACE	GAATTCTCTTAGTCACAATAC	ACTAACCCAAATCTACCATC
StPTB6 5' RACE	CCTTATGCTGGGCTGAACAT	AAAGGGCTGAAATAGCTGAA
StPTB6 3' RACE	ATGTACCCCTGGCAAGGTTCT	TCGTTTTCTCAGGTGCAAAGCATCTGAGA
StPTB6 5' RACE	CCTTATGCTGGGCTGAACAT	AAAGGGCTGAAATAGCTGAA
StPTB6 3' RACE	ATGTACCCCTGGCAAGGTTCT	TCGTTTTCTCAGGTGCAAAGCATCTGAGA
StGAI 5' RACE	CCATAGCCATCTCAAGTTG	GCTCATCCATTCCTGCATC
StGAI 3' RACE	CGATGGTTACAGAGTGGA	TTATAGCAACCTCCGCCT
**For Full-length cDNA**	**Forward primer (5' to 3')**	**Reverse primer (5' to 3')**
StPTB1	AAACCCTAGTCCCCCCTCTCCCTTTCCTCCATTT	TTTTTTTTTTACATAAGGAAAGCAG
Truncated StPTB1	AAACCCTAGTCCCCCCTCTCCCTTTCCTCCATTT	TTTTTTTTTTTTGTAAAGATCGACAAT
StPTB6	ATTCTATCTGGTCTTTCAAGCCCTAATT	TTTTTTAACAAACACAGAAGAAAGGAAGCA
Truncated StPTB6	ATTCTATCTGGTCTTTCAAGCCCTAATT	TTTTTTTTTTGAAAGAAAGAATCTTACATCTGGA
StPTB7	CCCAAAGGAAAGAAGCAGTACTCAAAGTCCGTTAA	TTTTTTTTTTGCAGATGAATCTTTTCCA
StGAI	GATATCTTCACGAGCACTTCT	TTTTTTTTTTCCCCAAACACCAAAT
**For Antisense/RNAi**	**Forward primer (5' to 3')**	**Reverse primer (5' to 3')**
PTB6AS	GAGCTCTTCATGTTCGCAACGTGGGCCA	ACTAGTTCCATATCCTTGTTGAGGAGATTTG
PTB6RNAi	CACCTTCATGTTCGCAACGTGGGCCA	TCCATATCCTTGTTGAGGAGATTTG

Table S2. Accession numbers of PTB proteins

Protein	Accession/Gene ID		Protein	Accession/Gene ID
HnRNP I	AAC99798.1		Barley1	TC217740
StPTB1	JF710641		Barley2	BAJ92028
StPTB6	JF710642		Barley3	BAJ94455
StPTB7	JF710643		Rice1	LOC_Os05g36120
TC201749	TC201749		Rice2	LOC_Os01g64770
CmRBP50	ACI43571		Rice3	LOC_Os03g25980
AtPTB1	At3g01150		Rice4	LOC_Os08g33830
AtPTB2	At5g53180		Rice5	LOC_Os01g43170

AtPTB3	At1g43190		Maize1	NP_001169470
Populus1	XP_002302167		Maize2	NP_001169809
Populus2	XP_002306709		Maize3	NP_001151769
Populus3	ABK95931		Maize4	NP_001137030
Populus4	XP_002324068		Soybean1	ACU20184
Populus5	ABK95840		Soybean2	TC392885
Populus6	XP_002322027		Soybean3	ACU23122
Sunflower1	TC53857		Soybean4	ACU18422
Sunflower2	TC47059		Medicago1	TC148772/Medtr1g098530.1
Tobacco1	TC84265		Medicago2	Medtr6g043520.1
Tobacco2	TC78548		Medicago3	Medtr6g012590.1
Tobacco3	TC80926		Medicago4	Medtr3g090960.1
Tobacco4	TC100085		Medicago5	Medtr7g011070.1
Tomato1	TC227660		Cucumber1	Csa005592
Tomato2	BF098030		Cucumber2	Csa015458
Tomato3	TC229790		Cucumber3	Csa009211
Tomato4	TC231069		StBEL5	AF406697
Wheat1	TC403305		StGAI	JF834157
Wheat2	TC377105		CmGAI	AY326306
Wheat3	TC386686		POTH1	U65648
Sorghum1	XP_002441164/09g021560		StGA2ox1	EU003995
Sorghum2	XP_002444395/07g021250		StGA20ox1	AJ291453
Sorghum3	XP_002467789/01g034060			

Table S3. Gene-specific primers used for RT-PCR.

Gene	Primer sequences (5' to 3')		Amplicon (bp)
	Forward primer	Reverse primer	
StPTB1	ATATGGAGACATGTACTCC	GGTTTATTGCGCAGAATC	246
StPTB6	TTTCCTCAGATGGGAAATGC	CTGGTCCGGTGTTAATATTT	331
StPTB7	CAACAGCAGTTCCGGTAC	ATTGCCTGATTAAGGTCA	188
StPTB1-1	GCCGTGGAGATGAGGTTTTCA	GGTCCATATAAATCAAGTGA	122
StPTB6-1	GAGGAGATGAGGTTTTCA	ATTTGCCCTTCGGCTCTGAT	212
StPTB6-2	TTTCAGAAGTCGGCCGGTT	AGAGAGATGCACACCTGA	393 (6-2.1), 314 (6-2.2)
StBEL5	GGGAGATTTTGGAAGGTTTG	TCAAATTGGGTCCTCCGACT	375
StGAI	AGCTGAAACAATCGGAGTTG	CATTTGAACCCAGATGAACC	527
18S	GGATGTTGCTTTTAGGACTC	CATCACAGACCTGTTATTGC	315
GA20ox1AS	GAGAAGCCTTGTGCCGTAGT	GAAGTCCGCCAACACAATCT	GA20ox1 Intron (WT: 649 bp, AS: 848 bp)

Table S4. Primers used for various constructs used in this report.

4-1. For cloning into pACT2 Yeast Protein Vector		
Gene	Forward Primer (5' to 3')	Reverse Primer (5' to 3')
CmRBP50	tacggatccacATGACTGAACCCTCAAAG (BamHI)	ctcgagctcTATACTCTGCAGCTGGGA (SacI)
StPTB1	tacggatccacATGTCTGATCCTTCAAA (BamHI)	cgatctcgagGATGCTCTGTAACTGAGA (XhoI)
StPTB6	tacggatccacATGACTGAGCCGTCAAAA (BamHI)	ctcgagctcGATGCTTTGCACCTGAGA (SacI)
StPTB7	tacggatccacATGTCAACATCCGGGCA (BamHI)	cgatctcgagACCATGATAAGGTGCAT (XhoI)
**4-2. For cloning into pIIIA/MS2-1 Yeast RNA vector**		
**Gene**	**Forward Primer (5' to 3')**	**Reverse Primer (5' to 3')**
CmGAI3	atcccccgggTCATCCTGACCCTT (SmaI)	cagcccgggATACATAAATCCTT (SmaI)
CmGAI0	atcccccgggATTTCACCGCGAAT (SmaI)	cagcccgggAAAGCCGGCCACTG (SmaI)
StBEL5 3'UTR	atcccccgggATACCAGAAAGTCTCGTA (SmaI)	cagcccgggGCTAATCTAATAATGATA (SmaI)
StBEL5 T1	atcccccgggACTCTTATATTGTG (SmaI)	cagcccgggAAAGAAGTATATAT (SmaI)
StBEL5 N	atcccccgggGAAGAGGTGGAGCA (SmaI)	cagcccgggTGTATGATTTGGCT (SmaI)
StGAI 3' UTR	atcccccgggTTATAAGTCAGCCT (SmaI)	cagcccgggTAAGATAAATAAAATA (SmaI)
**4-3. For promoter cloning into pBI101:GUS vector**		
**Gene**	**Forward primer (5' to 3')**	**Reverse primer (5' to 3')**
StPTB1	agtgtcgacGGTCTTGTCCCGACTTCCATC (SalI)	gtcggatccGGATCAGACAATTATACCTGAATCAC (BamHI)
StPTB6	agtgtcgacGCACAATAGCGATAAAATGACATAGTTC (SalI)	gtcggatccCGGCTCAGTCAATTTTTACCTGAATC (BamHI)
**4-4. For pBI121-GFP vector and 35S:StPTB-GFP constructs**		
**Gene**	**Forward primer (5' to 3')**	**Reverse primer (5' to 3')**
GFP (for pBI121-GFP)	tagaggatccATGGTGAGCAAGGGCGA (BamHI)	aattcgagctcTTACTTGTACAGCTCGT (SacI)
StPTB1	tactctagaATGTCTGATCCTTCAAA (XbaI)	cgatggatccGATGCTCTGTAACTGAGA (BamHI)
StPTB6	tactctagaATGACTGAGCCGTCAAAA (XbaI)	cgatggatccACCATGATAAGGTGCAT (BamHI)
StPTB7	tactctagaATGTCAACATCCGGGCA (XbaI)	cgatggatccACCATGATAAGGTGCAT (BamHI)

Table S5. Primers for quantitative RT-PCR, RIP and mobility assay (RMA)

Name	Primer sequence	Purpose
StBEL5F	CTGCAACAGCTAGGAATGATG	*StBEL5* real-time PCR primer (amplicon 137 bp)
StBEL5R	ATGATTTTGTCTGAATCCTTTGGG	
Ga2Ox1F	AGTCCCACCTGATCCCTAC	*Ga2Ox1* real-time PCR primer (amplicon 143 bp)
Ga2Ox1R	GCCCTCCAAAGTAAACCATTG	
StPTB1P	GCGCAATAAACCAGATCATGC	*StPTB1* real-time PCR primer (amplicon 112 bp)
StPTB1R	GTTGACTTCCAGACGCTTTTC	
StPTB6F	TCATGCTCTTGTTCAGATGGG	*StPTB6* real-time PCR primer (amplicon 150 bp)
StPTB6R	TGGTCCGGTGTTAATATTTGGG	
StPIN1F	GCACCAAATCCTGGCATGT	*StPIN1* real-time PCR primer (amplicon 63 bp)
StPIN1R	AGCTGTATTCTTGTGTGCTTTGGT	
StPIN4F	GTTTCATTGCGGCGGATTC	*StPIN4* real-time PCR primer (amplicon 60 bp)
StPIN4R	CCCATAGCGAAAGAACAACC	
B5H-F (w *Mlu*I)	tctACGCGTTTCTCAAAGCTTAGAGAGC	Construction of PVX/B5H for RMA of *StBEL5*
B5H-R (w *Eco*RV)	tctGATATCGTGGTGATGGTGATGATGGCTAATCTAATAATGATAGCAC	
B14H-F (w MluI)	tctACGCGTGATCATGGTCCTTCGTCTTCTAAG	Construction of PVX/B5H for RMA of *StBEL14*
B14H-R (w EcoRV)	tctGATATCGTGGTGATGGTGATGATGTATCATCATTCATCACCAATATATATTA	
GFP-F	ATGAGTAAAGGAGAAGAACTTTTCACTGGAG	GFP RT-PCR primer for RMA (Li et al, 2009)
GFP-R	TTTGTGTCCAAGAATGTTTCCATCTTC	
PVX-F	CAAGGTGCGCGAGGTTTACCAATC	PVX RT-PCR primer for RMA (Li et al, 2009)
PVX-R	GTATGCTGTTTCCGTTGTGATCTCTGTGAG	
B5HNP2F	GTTGTGATATTGTTCCTCTCAATTTGC	Real-time PCR of B5H for RMA (amplicon 151 bp)
B5HNP2R	ATCGTGGTGATGGTGATGATGGCT	
B14HF	TGGATAGTGAAAATCAGAATTTGCC	Real-time PCR of B14H for RMA (amplicon 142 bp)
B14HR	ATCGTGGTGATGGTGATGATGTATCATC	
18SF	GGGCATTCGTATTTCATAGTCAGAG	18S rRNA RT-PCR primer (Nicot et al, 2005)
18SR	CGGTTCTTGATTAATGAAAACATCCT	
StAct8F	GGAAAAGCTTGCCTATGTGG	Actin8 real-time PCR primer (Hannapel et al, 2013)
StAct8R	CTGCTCCTGGCAGTTTCAA	

RIPB22F	AACATTTGGGACCACTACGG	BEL22 RIP qRT-PCR
RIPB22R	ATCATAATTTATGTAGCAGTGTTTGAATAC	BEL22 RIP qRT-PCR
RIPB5R	ATTTTGTTACTTTAATTTAGTCTCCACTTC	BEL5 RIP qRT-PCR
RIPB5F	TCTTATATTGTGTGAGGCCTTCTG	BEL5 RIP qRT-PCR
BEL5GSP2	GTGATATTGTTCCTCTCAATTTGCA	GSP for heterograft RNA assay
NT-2	GCAACAGGATTCAATCTTAAGAAACT	GSP for heterograft RNA assay
